# A phase transition reduces the threshold for nicotinamide mononucleotide-based activation of SARM1, an NAD(P) hydrolase, to physiologically relevant levels

**DOI:** 10.1016/j.jbc.2023.105284

**Published:** 2023-09-22

**Authors:** Janneke Doedée Icso, Paul Ryan Thompson

**Affiliations:** 1 Program in Chemical Biology, University of Massachusetts Chan Medical School, Worcester, Massachusetts, USA; 2 Department of Biochemistry and Molecular Biotechnology, University of Massachusetts Chan Medial School, Worcester, Massachusetts, USA

**Keywords:** SARM1, phase transition, NMN, axon degeneration, hydrolase

## Abstract

Axonal degeneration is a hallmark feature of neurodegenerative diseases. Activation of the NAD(P)ase sterile alpha and toll-interleukin receptor motif containing protein 1 (SARM1) is critical for this process. In resting neurons, SARM1 activity is inhibited, but upon damage, SARM1 is activated and catalyzes one of three NAD(P)^+^ dependent reactions: (1) NAD(P)^+^ hydrolysis to form ADP-ribose (ADPR[P]) and nicotinamide; (2) the formation of cyclic-ADPR (cADPR[P]); or (3) a base exchange reaction with nicotinic acid (NA) and NADP^+^ to form NA adenine dinucleotide phosphate. Production of these metabolites triggers axonal death. Two activation mechanisms have been proposed: (1) an increase in the nicotinamide mononucleotide (NMN) concentration, which leads to the allosteric activation of SARM1, and (2) a phase transition, which stabilizes the active conformation of the enzyme. However, neither of these mechanisms have been shown to occur at the same time. Using *in vitro* assay systems, we show that the liquid-to-solid phase transition lowers the NMN concentration required to activate the catalytic activity of SARM1 by up to 140-fold. These results unify the proposed activation mechanisms and show for the first time that a phase transition reduces the threshold for NMN-based SARM1 activation to physiologically relevant levels. These results further our understanding of SARM1 activation and will be important for the future development of therapeutics targeting SARM1.

Neurodegenerative diseases, peripheral neuropathies, and traumatic axonal injuries encompass a wide range of neurological ailments that cause an irreversible loss of brain and/or motor function. A major feature of all neurodegenerative diseases is axonal degeneration. Axonal integrity is controlled by the levels of NAD^+^. In turn, NAD^+^ levels are controlled by nicotinamide nucleotide adenylyltransferase 2 (NMNAT2) and sterile alpha and toll/interleukin receptor (TIR) motif containing protein 1 [SARM1; (1)]. NMNAT2 synthesizes NAD^+^ from ATP and nicotinamide mononucleotide (NMN). By contrast, SARM1 hydrolyzes NAD^+^ to form ADP-ribose [ADPR; Fig. 1, *A* and *B*; (2, 3)]. SARM1 additionally catalyzes a cyclization reaction that produces cyclic-ADP-ribose (cADPR) and a base exchange reaction with NAD^+^-phosphate (NADP^+^) and NA to produce NA adenine dinucleotide phosphate [NAADP; Fig. 1*C*; (2, 3, 4, 5, 6)]. ADPR, cADPR, and NAADP are all potent inducers of calcium signaling (7, 8, 9, 10). Increased calcium influx activates calpains which degrade axonal components to promote degeneration (11). The loss of NAD^+^ also results in an energy crisis that can promote neuronal cell death (12). Notably, SARM1 knockout prevents neuronal cell death in response to injury and is protective in animal models of traumatic axonal injury, Alzheimer's disease, and chemotherapy-induced and diabetic peripheral neuropathies. As such, SARM1 is an attractive therapeutic target (13, 14, 15, 16, 17, 18, 19, 20, 21).

**Figure 1:**
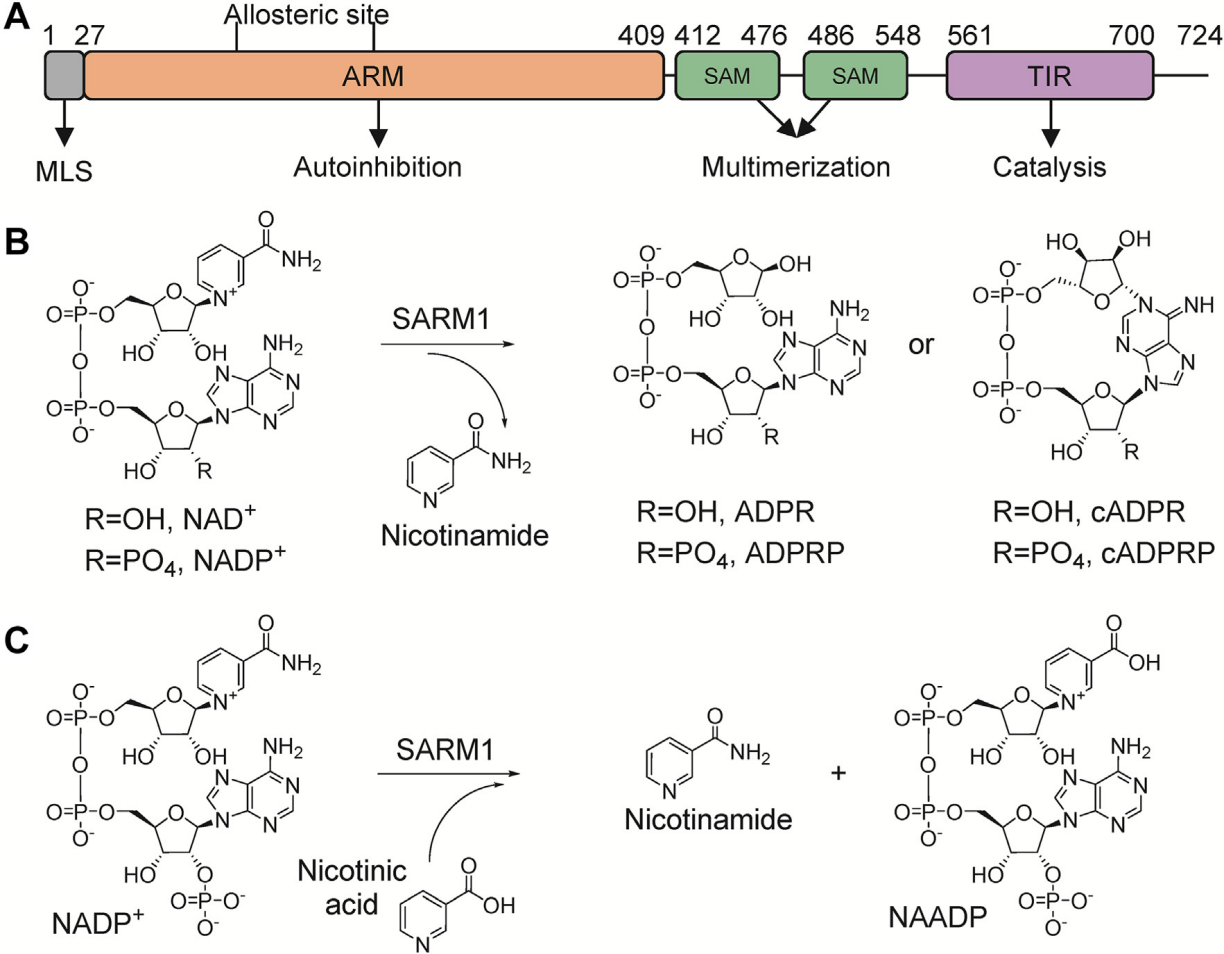
**SARM1 catalyzes multiple reactions.***A*, domain architecture of SARM1. *B*, schematic of NAD(P)^+^ hydrolysis and cyclization reactions catalyzed by SARM1. *C*, schematic of the base exchange reaction catalyzed by SARM1. SARM1, sterile alpha and toll-interleukin receptor motif containing protein 1.

Under healthy conditions, NMNAT2 is transported down the axon to facilitate localized NAD^+^ production. The NAD^+^ thus produced binds an allosteric pocket in the ARM domain of SARM1 with a dissociation constant (*K*_d_) of 24 μM to maintain the enzyme in an autoinhibited state (22). During axonal injury, NMNAT2 levels fall precipitously with a consequent decrease in NAD^+^ concentration and a rise in NMN levels (23, 24, 25). Under these conditions, NAD^+^ dissociates from the allosteric site and NMN binds in its place, causing a conformational change that activates SARM1 (4, 25, 26, 27). A role for NMN as a regulator of SARM1 activity is supported by *in vitro* kinetic data showing that high levels of NMN (>100 μM) upregulate SARM1 activity by 3-4-fold (4, 5, 26). In neurons, SARM1 activity can be modulated by the overexpression of NMN synthesizing enzymes, which increase the NMN/NAD^+^ ratio >10-fold (25, 28, 29).

We also showed that a liquid-to-solid phase transition potentiates the activity of the catalytic domain of SARM1 by >2000-fold. Although the mechanism by which PEG or citrate cause SARM1 to undergo a phase transition remains unknown, these agents likely act through a combination of excluded volume effects and changes in protein solvation that ultimately precipitate the enzyme (30, 31, 32). Notably, TIR-1, the *Caenorhabditis* *elegans* ortholog of SARM1, forms puncta *in vivo* and TIR-1 puncta size increases when axonal degeneration is triggered (18, 33, 34). TIR-1 activity also initiates p38 MAPK signaling in the context of intestinal immunity. Here, TIR-1 puncta form upon infection, resulting in the downstream activation of the PMK-1 MAPK pathway. These data show that SARM1 activation is associated with a phase transition *in vivo* in multiple contexts. Notably, the NMN-induced and phase transition-induced activation mechanisms are not mutually exclusive. Here, we provide evidence that the phase transition lowers the NMN concentration required to activate SARM1 by up to 140-fold. These data directly link these two activation mechanisms for the first time.

## Results

### Near full-length SARM1 undergoes a phase transition

We previously expressed and purified near full-length SARM1 (35). This construct lacks the first 27 amino acids, which encode an N-terminal mitochondrial localization sequence (MLS; SARM1^ΔMLS^). Using this construct, we recently showed that SARM1-mediated catalysis involves the formation of an oxocarbenium-like intermediate that is common to the hydrolysis, cyclization, and base exchange reactions (35). To further characterize SARM1 activity and regulation, we first defined the pH optimum. Briefly, SARM1^ΔMLS^ was incubated with NAD^+^ (100 μM) in reaction buffers ranging from pH 4.5 to 9. Since the NAD^+^ concentration is subsaturating (*K*_*m*_ = 30–50 μM with respect to NAD^+^, (4)), the pH profiles mimic *k*_*cat*_/*K*_m_ conditions. NAD^+^ depletion and product formation were monitored by HPLC. For NAD^+^ hydrolysis and cyclization, the pH optimum was 7.5 (Figs. S1*A* and S2, *A* and *B*). NADP^+^ hydrolysis likewise showed peak activity at pH 7.5 (Figs. S1*B* and S2, *C* and *D*). The pH optimum of the base exchange reaction between NADP^+^ and NA occurred at pH 5.5 (Figs. S1*B* and S2, *C* and *D*). These data are consistent with recently published pH profiles using the TIR domain of TIR-1 (34). Moreover, the base exchange reaction has been shown to occur in neurons, even though the pH optimum for this reaction is 5.5 (4).

**Figure 2:**
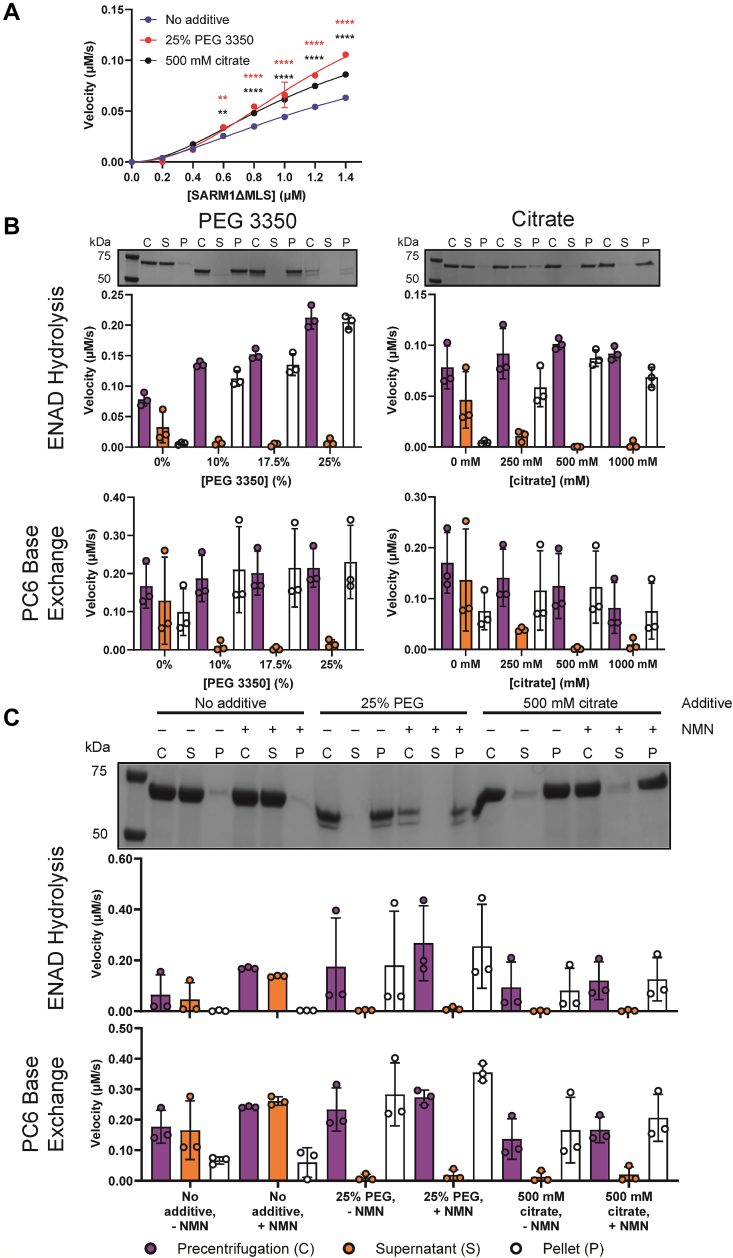
**SARM1**^**ΔMLS**^**undergoes a phase transition that correlates with enzyme activity.***A*, dose response of SARM1^ΔMLS^ ENAD hydrolysis activity in the presence or absence of 25% PEG3350 or 500 mM citrate; n = 3. Two-way ANOVA where ∗∗ = *p* > 0.01 and ∗∗∗∗ = *p* > 0.0001. *B*, SARM1^ΔMLS^ undergoes a phase transition that correlates with catalytic activity; n = 3. *C*, effect of NMN on the phase transition of SARM1^ΔMLS^; n = 3. The error is reported as SD for all graphs in this figure, though the error was smaller than the size of the data point and is not visible in some cases. ENAD, etheno-NAD^+^; NMN, nicotinamide mononucleotide; SARM1, sterile alpha and toll-interleukin receptor motif containing protein 1.

Having determined the optimal pH, we next evaluated the effect of crowding agents (*i.e.*, PEG3350 or citrate) on SARM1 activity. We previously showed that PEG and citrate induce a liquid-to-solid phase transition that increases the activity of the human and *C. elegans* TIR domain by 2000-and 30-fold, respectively (33, 34). SARM1^ΔMLS^ was incubated with PEG3350 and citrate and enzyme activity was evaluated using etheno-NAD^+^ (ENAD). ENAD is an NAD^+^ analog that fluoresces upon nicotinamide cleavage (36). At the highest enzyme concentration, hydrolysis activity increased 2-fold in PEG and 1.5-fold in citrate. The effect of these two additives was smaller at lower enzyme concentrations (Fig. 2*A*).

These data indicate that the effect of PEG and citrate on SARM1 activity is diminished relative to the TIR domain alone. This is expected because the SAM domains preorganize the TIR domains into a supramolecular structure [Fig. 1*A*; (27)]. Next, we determined whether SARM1^ΔMLS^ undergoes a phase transition. For these studies, SARM1^ΔMLS^ was incubated with 0 to 25% PEG or 0 to 1000 mM citrate, centrifuged, and resuspended. All fractions were analyzed by SDS-PAGE (Figs. 2*B* and S3*A*). If a phase transition does not occur, then we expect an intense band in the supernatant and no band or a faint band in the pellet. On the other hand, if the phase transition does occur, then we expect no band or a faint band in the supernatant and an intense band in the pellet. The control fraction does not undergo centrifugation, so an intense protein band is anticipated.

SARM1^ΔMLS^ was primarily located in the supernatant fraction in the no additive conditions. At all PEG percentages, the enzyme was found in the pellet fraction. It should be noted that PEG interferes with the ability of the protein to enter the gel, resulting in faint bands overall, and produces a double-band artifact at high concentrations. In citrate, the protein was split between the supernatant and pellet fractions at 250 mM, and predominantly in the pellet for the remaining citrate concentrations.

All fractions were also analyzed for catalytic activity in two fluorescent kinetic assays (1): the ENAD assay described above (34, 37), and (2) a base exchange assay that uses NAD^+^ and PC6 as substrates. In the latter assay, the base exchange product PC6 adenine dinucleotide (PAD6) is fluorescent (6). Notably, enzymatic activity in these assays corresponded to the location of the protein in the gel (Figs. 2*B* and S3*A*). These results are consistent with our previous results with the catalytic domain and show for the first time that near full-length SARM1 undergoes a phase transition *in vitro*. Moreover, these data validate our *in vivo* studies, which showed that full-length SARM1 also undergoes a liquid-to-solid phase transition that is associated with an increase in activity (33, 34).

In neurons, SARM1 activity is regulated by an increase in the concentration of NMN. However, as Freeman has noted, the concentration of NMN required for activation is supra-physiological (38). Hypothesizing that NMN may modulate the phase transition; we evaluated the effect of NMN on this transition using the same gel-based assay. The protein was located principally in the supernatant fraction in the no additive condition, whereas the protein was in the pelleted fraction when treated with PEG/citrate (Figs. 2*C* and S3*B*). Enzymatic activity again correlated with the location of the protein (Fig. 2*C*). Thus, NMN has no effect on the phase transition.

### A phase transition decreases the concentration of NMN required to activate SARM1

**Figure 3:**
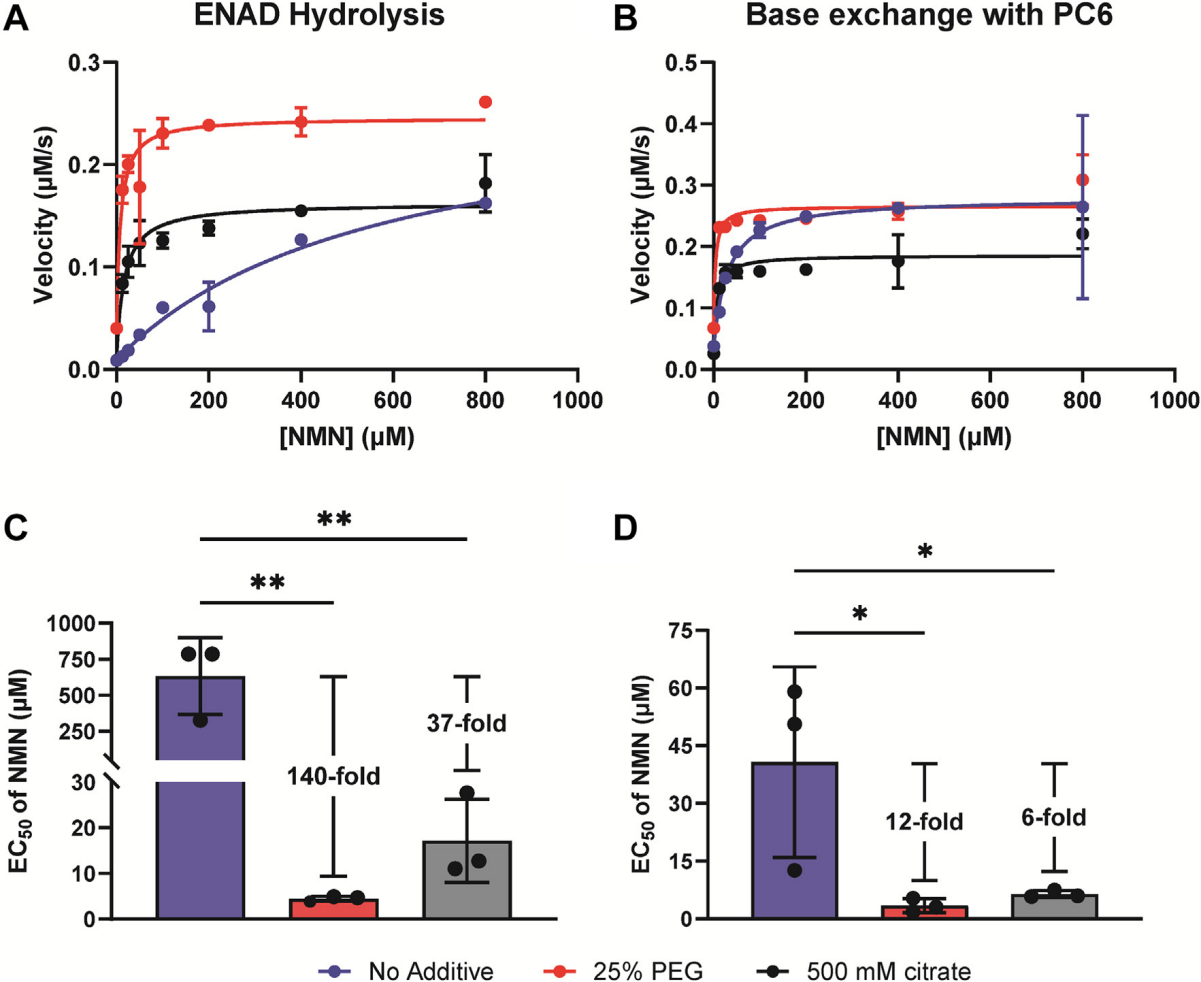
**The phase transition decreases the EC**_**50**_**of NMN to physiological concentrations.***A*, EC_50_ curves for NMN in the ENAD (1 mM) hydrolysis reaction in the presence or absence of 25% PEG3350 or 500 mM citrate; n = 3. *B*, EC_50_ curves for NMN in the base exchange reaction with NAD^+^ (1 mM) and PC6 (500 μM) in the presence or absence of 25% PEG3350 or 500 mM citrate; n = 3. *C*, bar graph of EC_50_s from A and S3C. *D*, bar graph of EC_50_s from *B* and S3*C*. For (*C* and *D*), two-way ANOVA where ∗ = *p* < 0.05 and ∗∗ = *p* < 0.01. The error is reported as SD for all graphs in this figure, though the error was smaller than the size of the data point and is not visible in some cases. ENAD, etheno-NAD^+^; NMN, nicotinamide mononucleotide; PC6, pyridyl conjugate 6.

Hypothesizing that the phase transition could lower the threshold for the NMN-induced activation of SARM1, we tested their effects in combination. The EC_50_ for NMN in the absence of additives was 570 μM for the hydrolysis reaction and 30 μM for the base exchange reaction. When the same EC_50_ assay was performed in the presence of PEG or citrate, the EC_50_ decreases. With PEG, the EC_50_ values for the hydrolysis and the base exchange reactions were decreased by 140-fold and 12-fold to 4.5 and 3.0 μM, respectively. The maximum velocity also increased 1.6-fold in the hydrolysis reaction. For citrate, the EC_50_ values were also decreased by 37-fold and 6-fold to 17 μM and 6.4 μM, respectively (Fig. 3, *A* and *B* and S3*C*). The maximum velocities remained similar with this crowding agent. Note that the substrate concentrations in these assays are above their *K*_*m*_ (see below). Together, these data indicate that the phase transition lowers the NMN concentration required to activate SARM1 hydrolysis by up to 140-fold (Fig. 3*C*) and the base exchange reaction by up to 12-fold (Fig. 3*D*). Of note, these data definitively link these two activation mechanisms for the first time.

### NMN and the phase transition do not affect SARM1 substrate specificity

Next, we evaluated the combined effect of NMN and PEG (or citrate) on the product specificity of the reaction (*i.e.*, the relative production of ADPR, cADPR, and NAADP). First, SARM1^ΔMLS^ was incubated with 100 μM NAD^+^ for 60 min in the presence or absence of PEG or citrate at 0 μM NMN, the EC_50_ for NMN, or 5x the EC_50_. Note that the NMN concentrations used here were based off EC_50_ values determined in the direct fluorescent assays (*i.e.*, 500 and 30 μM with no additive, 5 and 3 μM for assays in PEG, and 15 and 6 μM for assays with citrate for the hydrolysis and base exchange assays, respectively). The NMN concentrations in PEG and citrate are close to the physiological concentration of NMN (39, 40).

**Figure 4:**
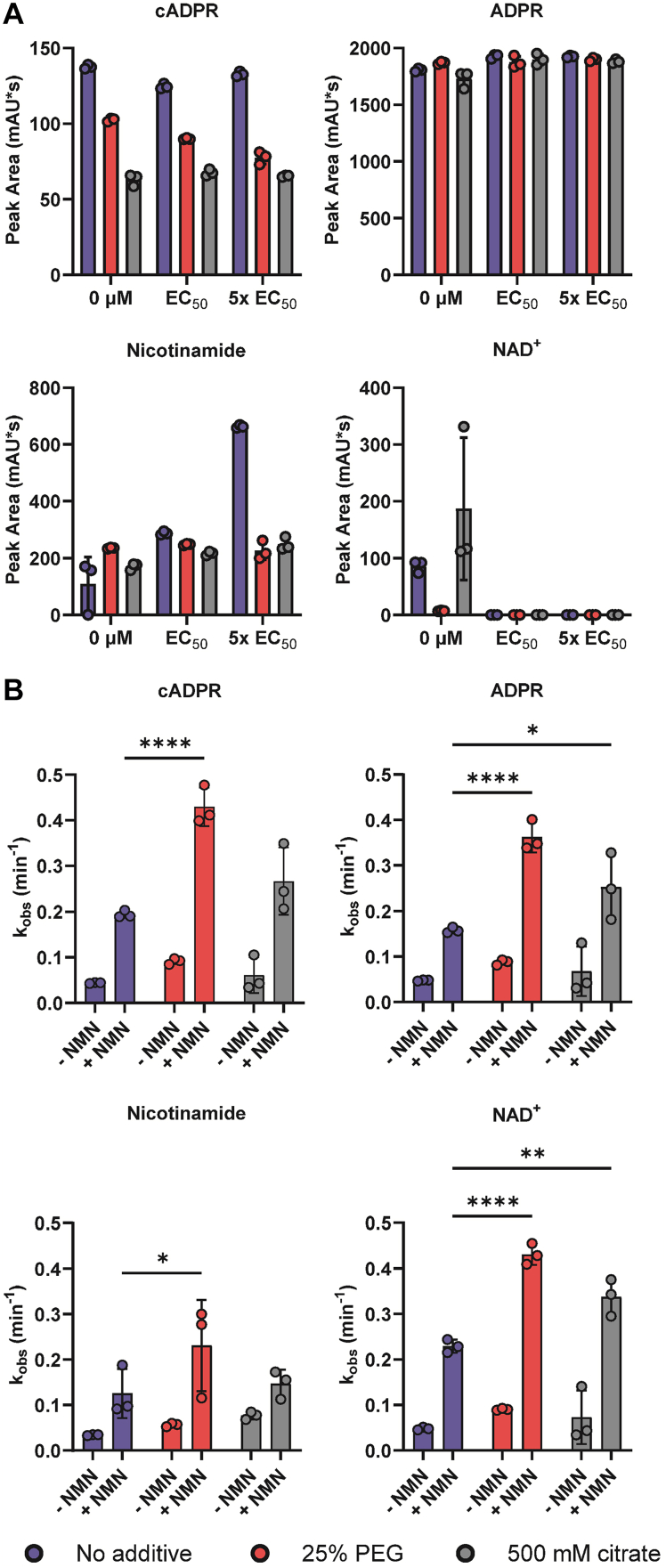
**NMN and the phase transition do not alter SARM1**^**ΔMLS**^**product specificity.***A*, product specificity of NAD^+^ hydrolysis after 60 min incubation in the presence or absence of 25% PEG3350 or 500 mM citrate at 0 μM NMN, NMN at the EC_50_, and 5x EC_50_; n = 3, error reported as SD. *B*, rates of NAD^+^ hydrolysis and cyclization in the presence or absence of 25% PEG3350 or 500 mM citrate at 0 μM NMN and at EC_50_; n = 3, error reported as SD. Two-way ANOVA where ∗ = *p* < 0.05, ∗∗ = *p* < 0.01, ∗∗∗ = *p* < 0.001, and ∗∗∗∗ = *p* < 0.0001. NMN, nicotinamide mononucleotide; SARM1, sterile alpha and toll-interleukin receptor motif containing protein 1.

When product levels were monitored by HPLC, only minor effects on the product specificity were observed. For example, there was no difference in ADPR or nicotinamide production across these conditions (Figs. 4*A* and S4, *A* and *B*). The apparent increase in nicotinamide production at 5× the NMN EC_50_ can be explained by NMN cleavage by SARM1^ΔMLS^ at the high NMN concentration used in this condition (2.5 mM; Fig. S5, *A*–*C*). In PEG, a slight NMN-dose-dependent decrease in cADPR production was observed (Figs. 4*A* and S4, *A* and *B*). This decrease likely reflects hydrolysis of cADPR by SARM1 (Fig. S5, *D*–*F*). The difference in scales for cADPR and ADPR reflects that SARM1 produces approximately 90% ADPR and 10% cADPR when hydrolyzing NAD^+^ (4). Likewise, we evaluated the product specificity of the base exchange reaction at pH values of 5.5 (optimum for the base exchange reaction) and pH 7.5 (optimum for the overall reaction). The analysis with 100 μM NADP and 1 mM NA showed that the combination of NMN and the phase transition did not alter the product specificity of the base exchange reaction either (Fig. S6, *A*–*D*).

### NMN and the phase transition increase the hydrolysis activity of SARM1

The effects of NMN and the phase transition on the rates of NAD^+^ hydrolysis and cyclization were also assessed. SARM1^ΔMLS^ was incubated with 100 μM NAD^+^ with and without NMN, quenched at specified time points from 0 to 60 min, and the reaction products analyzed by HPLC. The combination of NMN and PEG significantly increased NAD^+^ hydrolysis and cyclization 8-fold relative to the no additive, no NMN control. Citrate did not affect the cyclization rates compared to the no additive control but did increase the rate of NAD^+^ hydrolysis as measured by ADPR production and NAD^+^ consumption (Figs. 4*B* and S7, *A* and *B*).

Next, we evaluated the effect of NMN and the phase transition on the base exchange reaction rates. SARM1^ΔMLS^ was incubated with 100 μM NADP^+^ plus 1 mM NA with and without NMN, quenched at specified time points from 0 to 60 min, and analyzed by HPLC. The combination of NMN and the phase transition did not meaningfully affect reaction rates. (Fig. S7, *C*–*E*). Together these data show that NMN and the phase transition have minor effects on product specificity but increase the rates of the NAD^+^ hydrolysis and cyclization reactions instead.

**Figure 5:**
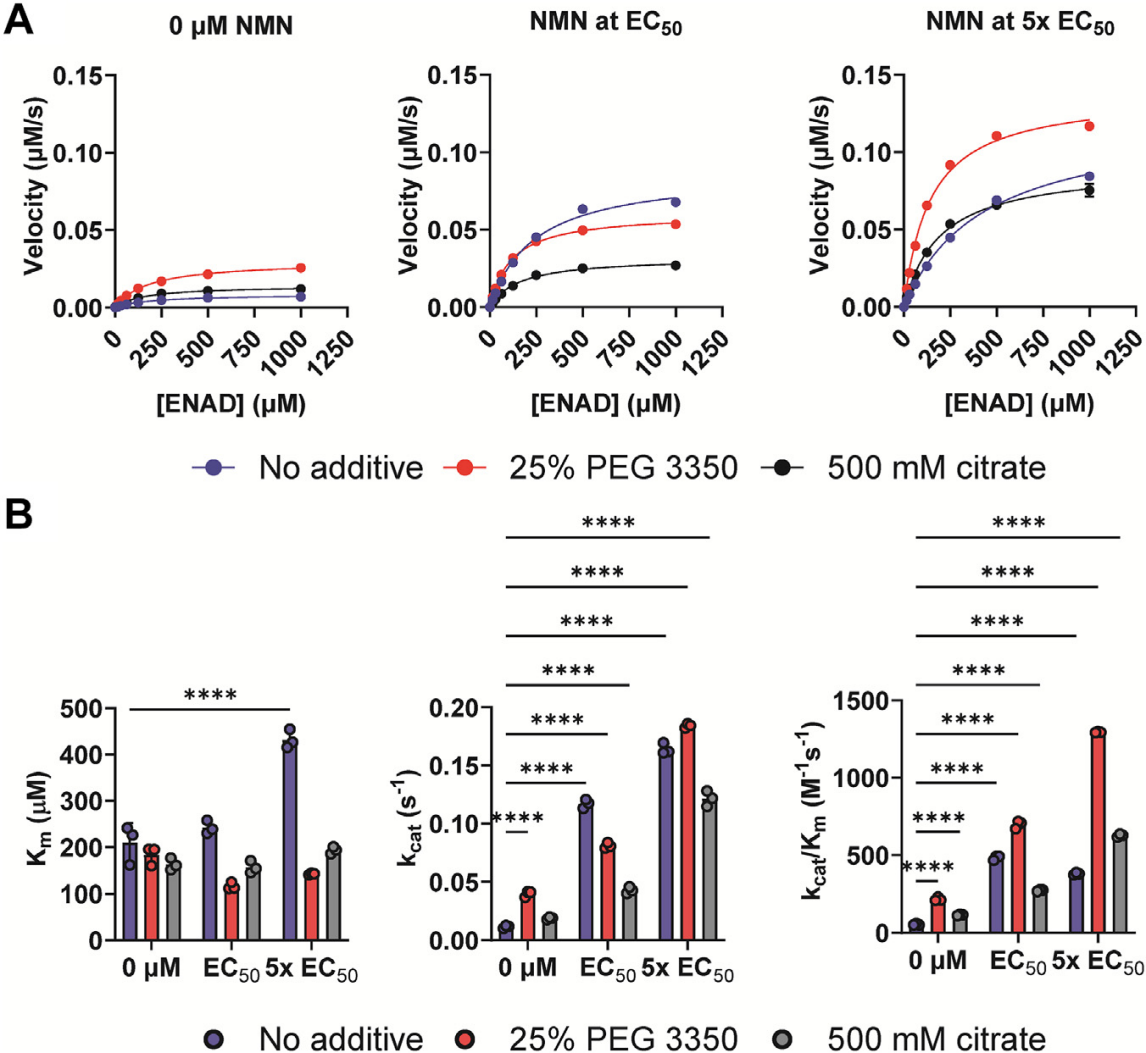
**NMN and the phase transition work additively to activate SARM1**^**ΔMLS**^**hydrolysis.***A*, kinetic analysis of SARM1^ΔMLS^ measured by ENAD hydrolysis in the presence or absence of 25% PEG3350 or 500 mM citrate at 0 μM NMN, NMN at the EC_50_, and 5x EC_50_; n = 3. *B*, kinetic parameters for ENAD hydrolysis in the presence or absence of 25% PEG3350 or 500 mM citrate at 0 μM NMN, NMN at the EC_50_, and 5x EC_50_. *K*_*m*_ (*left*), *k*_cat_ (*middle*), *k*_cat_/*K*_*m*_ (*right*); n = 3, two-way ANOVA. The error is reported as SD for all graphs in this figure, though the error was smaller than the size of the data point and is not visible in most cases. ENAD, etheno-NAD^+^; NMN, nicotinamide mononucleotide; SARM1, sterile alpha and toll-interleukin receptor motif containing protein 1.

Finally, we evaluated the effects of NMN on the kinetic parameters for NAD^+^ hydrolysis and base exchange in the direct fluorescent assays. For the hydrolysis reaction, we monitored ENAD hydrolysis in the presence and absence of PEG or citrate with 0-5x the EC_50_ μM NMN (Fig. 5). In the absence of crowding agents, the *K*_*m*_ for ENAD increased with increasing NMN concentration roughly 2-fold. Since NMN is a minor SARM1 substrate (Fig. S5, *A*–*C*), and the concentration used is 2.5 mM, it is possible that NMN competes for ENAD binding to the active site, accounting for this increase in *K*_*m*_. By contrast, in the presence of PEG or citrate, the *K*_*m*_ was relatively unchanged. The turnover number (*k*_*ca*t_) increased with increasing NMN levels by a maximum of 16-fold. The catalytic efficiency (*k*_*cat*_*/K*_*m*_) followed trends in *k*_*cat*_, increasing 23-fold maximally. Notably, the effect of NMN and crowding agents in combination was additive (Fig. 5, *A* and *B*). In the base exchange assay with NAD^+^ and PC6, minor differences were noted. In PEG, the *K*_*m*_ of PC6 increased significantly than the no additive controls; we hypothesize that this is an artifact of the nonphysiologic PC6 substrate, since NAD^+^ did not show this same increase in *K*_*m*_. *k*_*cat*_*/K*_*m*_ increased ∼2-fold maximally with NMN concentration. Crowding agents did not appear to activate the enzyme further in this assay (Fig. S8, *A*–*D*). Therefore, only NMN increases the base exchange activity, whereas NMN and the phase transition in PEG work additively to activate hydrolysis activity.

## Discussion

In summary, we show that a liquid-to-solid phase transition decreases the EC_50_ of NMN from 570 μM to as low as 4.5 μM for NAD^+^ hydrolysis and to 3.0 μM for the base exchange reaction. These lower values are consistent with physiological levels of NMN where its concentration is estimated to be ∼6 μM in brain tissue but as low as 10 to 20 nM in axons (39, 40). Thus, the phase transition modulates the ability of SARM1 to respond to cellular NMN levels. Note that the EC_50_ value for NMN in the absence of crowding agent measured here (*i.e.*, 570) is ∼10-fold higher than previously reported (∼50 μM). However, in that case, it is important to recognize that pure protein was not assayed. Instead, the authors used lysates from cells that express SARM1 (5). We and others have previously shown that SARM1 loses activity upon purification and the use of cell lysates appears to mimic the effects of crowding agents such as PEG and citrate (33, 37). In fact, most *in vitro* assays employ some type of crowding to observe activity (*e.g.*, on bead or in lysates; Table S1).

A recent cryo-EM structure of NMN bound to the ARM-SAM portion of human SARM1 showed that the ARM domain compacts upon the binding of NMN to the ARM domain (41). Because the linker between the ARM and SAM domains is rigid, the ARM domain rotates 22° and hinges upward from the SAM domains by 19Å. This movement causes steric clashes with the TIR domains that forces them to oligomerize. The activated TIR domains in SARM1 adopt a unique two-stranded antiparallel linear complex above the ARM–SAM ring. Moreover, 2-D class averages show that the TIR domain assembly is somewhat curved and that additional SARM1 octamers can append to either end of the linear TIR domain structure to extend the active formation (27, 41). In other words, SARM1 catalysis is associated with a change in conformation and supramolecular oligomeric state.

**Figure 6:**
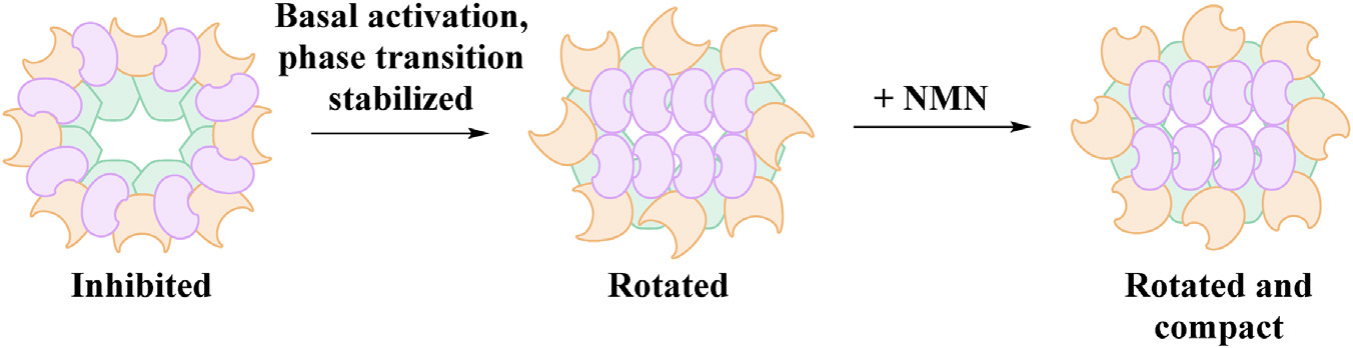
**Model by which the phase transition reduces the threshold for NMN based activation of SARM1.***Orange* = ARM domain; *green* = SAM domains; *purple* = TIR domains. NMN, nicotinamide mononucleotide; SARM1, sterile alpha and toll-interleukin receptor motif containing protein 1; TIR, toll/interleukin receptor.

Since SARM1 has a basal activity when NMN levels are low, the ARM domain can likely adopt this lifted rotated state even in the absence of NMN. However, the ARM domain is likely not as compacted as when NMN is bound. Consequently, we hypothesize that the phase transition stabilizes the lifted rotated supramolecular conformation of the enzyme, such that NMN serves to only compact the ARM domain upon binding instead of compacting, rotating, and lifting the ARM domain (Fig. 6). Functionally, this has the effect of making the enzyme more responsive to NMN and decreased the EC_50_ for NMN.

Since the experiments described above were completed *in vitro*, a limitation of our approach is that it does not explore how the phase transition may be modulated by proteins that interact with SARM1 (*e.g.*, TRIF, PINK1, and JNK), nor how post translational modifications affect the phase transition (42, 43, 44). In total, our results show for the first time that a phase transition reduces the threshold for the NMN induced activation of SARM1 to physiologically relevant levels. The phase transition likely promotes a conformation that facilitates NMN binding and SARM1 activation. This conclusion is consistent with the fact that there were only minor effects on the product specificity and kinetic parameters for the reactions. These data are also consistent with our *in vivo* data with full-length *C. elegans* SARM1 where we observe puncta formation upon axonal injury or in response to pathogen infection and provide a biological basis for this phenomenon.

## Experimental procedures

### SARM1^ΔMLS^ expression and purification

The expression and purification of SARM1^ΔMLS^ has been described recently (35). Briefly, the SARM1^ΔMLS^ expression construct codes for amino acids 28 to 724 of human SARM1, a PreScission Protease cut site, and two tandem Protein A tags cloned into the pcDNA3.4(+) vector with BspEI and EcoRV restriction sites. This vector was maintained as an XL1Blue *E. coli* (Agilent) glycerol stock at −80 °C. Plasmid was purified from 500 ml LB cultures supplemented with 100 μg/ml ampicillin and 10 μg/ml tetracycline according to the QIAGEN Plasmid Maxi Kit manufacture’s protocol. Sanger sequencing was used to verify the sequence of the plasmid (Azenta).

Expi293F cells growing in FreeStyle 293 Expression Medium (Thermo Fisher Scientific) were seeded at 2.5 to 3.0 × 10^6^ cells/ml and incubated for 18 h at 37 °C, 8% CO_2_, <80% relative humidity, and 125 rpm agitation. The next day, cells were diluted to 2.5 × 10^6^ cells/ml. Plasmid DNA (3 μg/ml of culture) and PEI-MAX (3 μg/μg of DNA) were diluted in Opti-MEM (Thermo Fisher Scientific). The Opti-MEM dilutions were incubated separately at room temperature for 5 min and then together for 30 min at room temperature. Next, the DNA:PEI mixture was added to the cultures at a final DNA concentration of 3 μg/ml and the cultures were incubated for 16 to 20 h. The day after transfection, cultures were diluted 1:1 with valproic acid supplemented media so that the final concentration of valproic acid was 2.2 mM. Cells were incubated for 72 h before harvesting by centrifugation at 500*g* for 5 min at room temperature. Cell pellets were flash frozen in liquid nitrogen and stored at −80 °C.

SARM1^ΔMLS^ was purified as previously described (35). Briefly, pellets from 300 ml cultures were thawed on ice and resuspended in 10 ml of lysis buffer (50 mM Hepes pH 8; 400 mM NaCl; 5% glycerol [w/v]). The cell suspension was sonicated in 4 ml batches with a Thermo Fisher Scientific Sonic Dismembrator sonicator (FB-705) for a total of 10 times at amplitude 10 for 10 s pulsing on and off for 1 s each, followed by a 20 s period between each sonication event. Crude lysate was clarified by centrifugation at 15,000*g* at 4 °C for 10 min. Rabbit IgG agarose resin (5 ml, Sigma-Aldrich) was equilibrated with lysis buffer. Supernatant from the clarification step was mixed with the equilibrated resin and bound batch-wise for 1 h at 4 °C using an end-over-end rocker. Material that did not bind to the resin was allowed to flow off the column by gravity. Resin was washed two times with ten column volumes (CV) of lysis buffer and 5 CV of wash buffer (25 mM Hepes pH 7.4; 150 mM NaCl). PreScission protease (250 μl) was diluted in 4.75 ml wash buffer and used to resuspend the resin. PreScission protease was incubated with the resin overnight at 4 °C without agitation. The next day, cleaved protein was allowed to flow off the column by gravity and the column was washed twice with 5 CV of wash buffer. Meanwhile, Glutathione Sepharose resin (1 ml; Sigma-Aldrich) was equilibrated with wash buffer. The eluate and first wash from the immunoglobulin G column were mixed with the Sepharose resin. Protein was allowed to flow by gravity. Protein purity was evaluated by SDS-PAGE (Fig. S9) and the pure protein was concentrated in an Amicon Centrifugal Filter Unit MWCO 10,000. Protein concentration was determined by the Bradford method. Aliquots (25 μl) were flash frozen in liquid nitrogen and stored at −80 °C.

### pH profiles for the hydrolysis, cyclization, and base exchange reactions

SARM1^ΔMLS^ (750 nM) was incubated for 60 min at 25 °C with 100 μM NAD^+^ in 50 mM buffer and 150 mM NaCl in triplicate. In parallel, SARM1^ΔMLS^ (750 nM) was incubated for 60 min at 25 °C with 100 μM NADP^+^ and 1 mM NA in 50 mM buffer and 150 mM NaCl in triplicate. For pH 4.5 and 5.0, sodium acetate buffer was used; Mes buffer was used for pH 5.5 to 6.5; and Tris was used for pH 7.0 to 8.0. The reactions were stopped by the addition of 0.25% TFA (final concentration) and stored at −20 °C until analysis. After thawing, the samples were filtered through a 0.22 μm Costar Spin-X cellulose acetate centrifugal filter prewashed with deionized water.

Samples prepared with NAD^+^ (50 μl) were injected onto a Supelcosil LC-18 column (5 μm, 4.6 × 250 mm; Supelco) attached to an Agilent 1260 HPLC. A gradient method was used to elute metabolites from the column as previously described (35): 100% A for 9 min, 0% B to 12% B for 6 min, 12% B to 45% B for 2.5 min, 45% B to 100% for 2.5 min, 100% B for 5.5 min, 100% B to 100% A for 5 min, and 100% A for 4.5 min at a flow rate of 1.3 ml/min. Potassium phosphate (100 mM) pH 6.0 comprised Buffer A; 100 mM potassium phosphate pH 6.0 with 20% MeOH comprised Buffer B (45). For samples prepared with NADP^+^ and NA, 25 μl was loaded onto a POROS HQ column (10 μm, 4.6 × 100 mm, 1.7 ml; Thermo Fisher Scientific) attached to an Agilent 1260 HPLC. Metabolites were eluted from the column using the following gradient: 100% buffer A for 1 min, 0% to 50% buffer B over 8.25 min, 50% to 100% buffer B over 3 min, 100% buffer B to 100% buffer A over 1 min, and 100% buffer A for 3 min at a flow rate of 1 ml/min. Buffer A consisted of 10 mM ammonium acetate pH 5.0 and buffer B consisted of 1 M ammonium acetate pH 5.0. Absorbances were collected at 254 nm and HPLC traces were plotted in GraphPad Prism (https://www.graphpad.com/features). Areas under the curve were calculated using ChemStation (Agilent) and plotted in GraphPad Prism.

### Effect of PEG 3350 and sodium citrate on the hydrolysis of ENAD

To generate a standard curve of EADPR (the product of the ENAD hydrolysis reaction), 400 μM nicotinamide 1,N^6^-ethenoadenine dinucleotide (ENAD, Sigma-Aldrich) was incubated with excess ADP-ribosyl cyclase (Sigma-Aldrich) in 20 mM Hepes pH 7.5 and 150 mM NaCl for 30 min in a Corning 96–well Half Area Black Flat Bottom Polystyrene NBS Microplate. Reaction progress was monitored by fluorescence (λ_ex_ = 340 nm, λ_em_ = 405 nm) every 60 s using the Wallac EnVision Manager software and a PerkinElmer EnVision 2104 Multilabel Reader (www.perkinelmer.com). When a plateau was observed, the reactions were serially diluted (1:2) with buffer for a total of 7 ENAD concentrations and a buffer blank and the fluorescence intensities of all samples were measured. Fluorescence intensities were plotted against ENAD concentration to generate the standard curve. The standard curve was generated in duplicate, and the slope of the line was averaged so that there was a single conversion factor.

To evaluate the effect of PEG or citrate on SARM1 hydrolysis activity, SARM1^ΔMLS^ (0–1.4 μM) was incubated in 20 mM Hepes pH 7.5 and 150 mM NaCl with and without 25% PEG 3350 or 500 mM sodium citrate for 10 min at room temperature in triplicate. The reaction was initiated with 1 mM ENAD and monitored for 15 min every 15 s at 25 °C using λ_ex_ = 340 nm/λ_em_ = 405. Fluorescence intensities were converted to concentration using the standard curve above. Velocities were determined from plots of product concentration *versus* time and plotted in GraphPad Prism 9. The data were fit to Equation 1:

(Eq. 1) $$Y=\frac{B_{\max}\times X^{h}}{{\left(K_{d}\right)}^{h}+X^{h}}$$

where B_max_ is the maximum specific binding, K_d_ is the concentration of enzyme needed to achieve half-maximal binding, and h is the Hill slope.

### SARM1^ΔMLS^ undergoes a phase transition

SARM1^ΔMLS^ (2.5 μM) was incubated in 20 mM Hepes pH 7.5 and 150 mM NaCl with 0 to 25% PEG 3350 or 1 to 1000 mM sodium citrate for 15 min at room temperature in duplicate. Samples were removed as the precentrifugation control, and the remaining sample was centrifuged at 21,000*g* for 10 min at 4 °C. Subsequently, the supernatant was removed, and the pellet fraction was resuspended in buffer with the corresponding concentration of additive. Protein content of the samples was evaluated by SDS-PAGE. Additionally, all fractions were analyzed for enzymatic activity in the ENAD and base exchange direct fluorescent assays (6, 35, 46). Samples were aliquoted into black microplates and reactions were initiated with 1 mM ENAD or 1 mM NAD^+^ and 500 μM PC6; PC6 was synthesized as previously described (6, 35, 46). Reactions were monitored for 20 min every 15 s at 25 °C using λ_ex_ = 340 nm/λ_em_ = 405 for the ENAD reaction and λ_ex_ = 390 nm/λ_em_ = 520 for the base exchange reaction with PC6. Fluorescence intensities were converted to concentration units using a standard curve; the method to generate the EADPR standard curve is described above, and the PAD6 standard curve is described below. Velocities were determined from plots of product concentration *versus* time; only data up to 10% substrate turnover were used in velocity calculations. Velocities were plotted in GraphPad Prism 9.

For the PAD6 standard curve, 400 μM PC6 and 2 mM NAD^+^ with excess TIR domain of TIR-1 in 50 mM Tris pH 8.0 and 150 mM NaCl in the presence of 25% PEG 3350 or 500 mM sodium citrate. The reaction was monitored for 1.5 h at 25 °C using λ_ex_ = 390 nm/λ_em_ = 520 on a PerkinElmer EnVision 2104 Multilabel Reader and Wallac EnVision Manager software. Reaction progress was monitored every min until a plateau was reached, at which point, a 1:2 serial dilution was performed with 50 mM Tris pH 8.0 and 150 mM NaCl in the presence of 25% PEG 3350 or 500 mM sodium citrate. After dilution, one reading was taken and these values were plotted in GraphPad Prism 9 to generate the standard curve. The standard curve was generated in duplicate, and the slope of the line was averaged so that there was a single conversion factor. The slope in both PEG and citrate was 25, and so we assumed that PEG or citrate did not affect PC6 or PAD6 itself.

### Effect of the phase transition on the EC_50_ of NMN

For the ENAD hydrolysis reaction, SARM1^ΔMLS^ (750 nM) was incubated for 10 min at room temperature with 0 to 800 μM NMN and in the presence or absence of 25% PEG 3350 or 500 mM sodium citrate in 20 mM Hepes pH 7.5 and 150 mM NaCl in triplicate. The reaction was initiated with 1 mM ENAD and monitored for 20 min at 25 °C every 15 s at λ_ex_ = 340 nm/λ_em_ = 405. For the base exchange reaction with PC6, SARM1^ΔMLS^ (750 nM) was incubated for 10 min at room temperature in 20 mM Hepes pH 7.5 and 150 mM NaCl with 500 μM PC6, 0 to 800 μM NMN, and in the presence or absence of 25% PEG 3350 or 500 mM sodium citrate in triplicate. The reaction was initiated with 1 mM NAD^+^ and monitored for 20 min at 25 °C every 15 s at λ_ex_ = 390 nm/λ_em_ = 520. Fluorescence intensities were converted to EADPR or PAD6 concentrations using the corresponding standard curves described above. Velocities were determined by calculating the slope from plots of product concentration *versus* time. Only data up to 10% substrate turnover were used in velocity calculations.

Initial fits of the data showed that some EC_50_ values for NMN were outside the original range of NMN concentrations. Therefore, these assays were repeated using a narrower range of NMN concentrations (0–100 μM *versus* 0–800 μM). Specifically, both the hydrolysis and base exchange reactions were repeated in PEG and the base exchange was repeated in citrate with this narrower range of NMN.

### Effect of NMN on the phase transition of SARM1^ΔMLS^

SARM1^ΔMLS^ (5 μM) was incubated in 20 mM Hepes pH 7.5 and 150 mM NaCl with or without 25% PEG or 500 mM sodium citrate with 0 μM NMN or NMN at the EC_50_ concentration; for the no additive condition, the NMN concentration was 500 μM, 5 μM in PEG, and 15 μM in citrate. A portion of the sample was removed for the precentrifugation control, and the remainder of the sample was centrifuged at 21,000*g* for 10 min at 4 °C. Following centrifugation, the supernatant and pelleted fractions were separated, and the pellet was resuspended in buffer with the corresponding additives and NMN concentration. All samples were assayed for enzymatic activity in the ENAD hydrolysis and PC6 base exchange assays as described above; reactions were initiated with 1 mM ENAD or 1 mM NAD^+^ and 500 μM PC6, respectively. Velocities were calculated from plots of product concentration *versus* time and plotted in GraphPad Prism 9; only data corresponding to up to 10% substrate turnover were used in calculations.

### Product specificity of the hydrolysis, cyclization, and base exchange reactions catalyzed by SARM1^ΔMLS^

For reactions with NAD^+^, 100 μM NAD^+^ and 750 nM SARM1^ΔMLS^ were mixed with 20 mM Hepes pH 7.5 and 150 mM NaCl in the presence or absence of 25% PEG 3350 or 500 mM sodium citrate and with or without NMN. The NMN concentrations were 500 μM (EC_50_) or 2.5 mM (5× the EC_50_) in the no additive condition, 5 μM (EC_50_) or 25 μM (5× the EC_50_) in PEG, and 15 μM (EC_50_) or 75 μM (5× the EC_50_) in citrate. Reactions were incubated for 60 min at 25 °C. The reactions were quenched by adding 0.25% TFA (final concentration) and stored at −20 °C until analysis. Samples were thawed and filtered through a 0.22 μm cellulose acetate centrifugal filter. Fifty microliters of the filtered samples were loaded onto a Supelcosil LC-18 column (5 μm, 4.6 × 250 mm; Supelco) attached to an Agilent 1260 HPLC. See Table S2 for solvent and gradient details.

For reactions with NADP^+^ and NA, 100 μM NADP^+^, 1 mM NA, and 750 nM SARM1^ΔMLS^ were mixed with 50 mM buffer (pH 5.5 [Mes] or 7.5 [Tris]) and 150 mM NaCl in the presence or absence of 25% PEG 3350 or 500 mM sodium citrate and with or without NMN. The NMN concentrations were 30 μM (EC_50_) or 150 μM (5× the EC_50_) in the no additive condition, 3 μM (EC_50_) or 15 μM (5× the EC_50_) in PEG, and 6 μM (EC_50_) or 30 μM (5× the EC_50_) in citrate. Reactions were incubated for 60 min at 25 °C. The reactions were quenched by adding 0.25% TFA (final concentration) and stored at −20 °C until analysis. Samples were thawed and filtered through a 0.22 μm cellulose acetate centrifugal filter. A portion of the sample (25 μl) was loaded onto a POROS HQ column (10 μm, 4.6 × 100 mm, 1.7 ml; Thermo Fisher Scientific) attached to an Agilent 1260 HPLC. See Table S2 for buffer and gradient details. For all samples, absorbance was collected at 254 nm and HPLC traces were plotted in GraphPad Prism 9. Areas under the curve were calculated using ChemStation (Agilent) and plotted in GraphPad Prism 9.

### Evaluating NMN and cADPR as SARM1^ΔMLS^ substrates

SARM1^ΔMLS^ (750 μM) was incubated with 2.5 mM NMN or 100 μM cADPR for 0, 5, 15, 30, and 60 at 25 °C in 20 mM Hepes pH 7.5 and 150 mM NaCl) in triplicate; the cADPR samples also contained 25% PEG 3350. The reactions were stopped at the specified times by adding 0.25% TFA (final concentration) and flash frozen in liquid nitrogen. Samples were thawed and filtered through a 0.22 μm cellulose acetate centrifugal filter. A portion of the filtered sample (50 μl) was loaded onto a Supelcosil LC-18 column (5 μm, 4.6 × 250 mm; Supelco) attached to an Agilent 1260 HPLC. See Table S2 for solvent and gradient details. Absorbance was collected at 254 nm and HPLC traces were plotted in GraphPad Prism 9. Areas under the curve were calculated using ChemStation (Agilent) and plotted in GraphPad Prism 9.

### Time course experiments for the reactions catalyzed by SARM1^ΔMLS^

For reactions with NAD^+^, 100 μM NAD^+^ and SARM1^ΔMLS^ (750 nM) were mixed in 20 mM Hepes pH 7.5 and 150 mM NaCl in the presence or absence of 25% PEG 3350 or 500 mM sodium citrate and with or without NMN. The NMN concentrations were 2.5 mM in the no additive condition, 25 μM in PEG, and 75 μM in citrate (*i.e.* 5× the EC_50_). Reactions were incubated at 25 °C and quenched by adding 0.25% TFA (final concentration) at 0, 5, 15, 30, and 60 min. Samples were stored at −20 °C until analysis, and, upon thawing, filtered through a 0.22 μm cellulose acetate centrifugal filter. The filtered sample (50 μl) was loaded onto a Supelcosil LC-18 column (5 μm, 4.6 × 250 mm; Supelco) attached to an Agilent 1260 HPLC. See Table S2 for solvent and gradient details.

For reactions with NADP^+^ and NA, 100 μM NADP^+^, 1 mM NA, and 750 nM SARM1^ΔMLS^ was mixed with 50 mM Mes pH 5.5 and 150 mM NaCl in the presence or absence of 25% PEG 3350 or 500 mM sodium citrate and with or without NMN. The NMN concentrations were 150 μM in the no additive condition, 15 μM in PEG, and 30 μM in citrate (*i.e.*, 5× the EC_50_). Reactions were incubated at 25 °C and quenched at 0, 5, 15, 30, and 60 min by adding 0.25% TFA (final concentration). Samples were filtered through a 0.22 μm cellulose acetate centrifugal filter. A portion of the sample (25 μl) was loaded onto a POROS HQ column (10 μm, 4.6 × 100 mm, 1.7 ml; Thermo Fisher Scientific) attached to an Agilent 1260 HPLC. See Table S2 for buffer and gradient details. For all samples, absorbance was collected at 254 nm and HPLC traces were plotted in GraphPad Prism 9. Areas under the curve were calculated using Chem Station (Agilent) and plotted in GraphPad Prism 9. Data were fitted to the equation for one-phase decay to determine *k*_*obs*_.

### Evaluation of the effect of NMN on the kinetics of SARM1^ΔMLS^

For the ENAD hydrolysis assay, 750 nM SARM1^ΔMLS^ was incubated in 20 mM Hepes pH 7.5 and 150 mM NaCl in the presence or absence of 25% PEG 3350 or 500 mM sodium citrate and with or without NMN. The NMN concentrations were 500 μM (EC_50_) or 2.5 mM (5× the EC_50_) in the no additive condition, 5 μM (EC_50_) or 25 μM (5× the EC_50_) in PEG, and 15 μM (EC_50_) or 75 μM (5× the EC_50_) in citrate. The mixtures were incubated at room temperature for 10 min and then reactions were initiated with 0 to 1000 μM ENAD. Reaction progress was monitored for 20 min every 15 s at 25 °C using λ_ex_ = 340 nm/λ_em_ = 405. Fluorescence intensities were converted to concentration using the EADPR standard curve described above.

For the base exchange assay with PC6, NAD^+^ was titrated while holding the PC6 concentration constant and PC6 was titrated while the NAD^+^ concentration was held constant. To titrate NAD^+^, 750 nM SARM1^ΔMLS^ and 500 μM PC6 were incubated in 20 mM Hepes pH 7.5 and 150 mM NaCl in the presence or absence of 25% PEG 3350 or 500 mM sodium citrate and with or without NMN. The NMN concentrations were 30 μM (EC_50_) or 150 μM (5× the EC_50_) in the no additive condition, 3 μM (EC_50_) or 15 μM (5× the EC_50_) in PEG, and 6 μM (EC_50_) or 30 μM (5× the EC_50_) in citrate. The mixtures were incubated at room temperature for 10 min and then reactions were initiated with 0 to 1000 μM NAD^+^. To titrate PC6, 750 nM SARM1^ΔMLS^ and 0 to 500 μM PC6 were mixed in the same reaction conditions and the reaction was initiated with 1 mM NAD^+^. Reaction progress was monitored for 20 min every 15 s at 25 °C using λ_ex_ = 340 nm/λ_em_ = 405. Fluorescence intensities were converted to concentration using the PAD6 standard curve described above.

Velocities were determined by calculating the slope from the product concentration *versus* time plots and these values were plotted in GraphPad Prism 9. Only data corresponding to up to 10% substrate turnover were used in velocity calculations. Data were fit to the Michaelis–Menten equation and bar graphs of the kinetic parameters (*i.e.*, *K*_*m*_, *k*_cat_, *k*_cat_/*K*_*m*_) were generated.

## Data availability

Further information and requests for resources and reagents should be directed to and will be fulfilled by P. R. T.

## Supporting information

This article contains supporting information pertaining to: Figs. S1–S9 depicting full HPLC chromatographs, full gel images, and additional kinetic data; and Tables S1 and S2 describes assay conditions used in the literature (references S1–S25) and HPLC solvents.

## Conflict of interest

The authors declare that they have no conflicts of interest with the contents of this article.
