# Supplementary material for: A phase transition reduces the threshold for nicotinamide mononucleotide-based activation of SARM1, an NAD(P) hydrolase, to physiologically relevant levels
Source: J Biol Chem. 2023 Sep 22;299(11):105284. doi: 10.1016/j.jbc.2023.105284 (PMC10624580; doi:10.1016/j.jbc.2023.105284)
Supplement: Supporting Figures S1–S9 and Tables S1 and S2 [file mmc1.pdf]

Supporting Information for

"A phase transition reduces the threshold for nicotinamide mononucleotide-based activation of SARM1, an NAD(P) hydrolase, to physiologically relevant levels"

Janneke D. Icso[1,2] and Paul R. Thompson[1,2*]

[1]Program in Chemical Biology, University of Massachusetts Chan Medical School, Worcester, MA, 01605

[2]Department of Biochemistry and Molecular Biotechnology, University of Massachusetts Chan Medial School, Worcester, MA, 01605

[*]Corresponding author

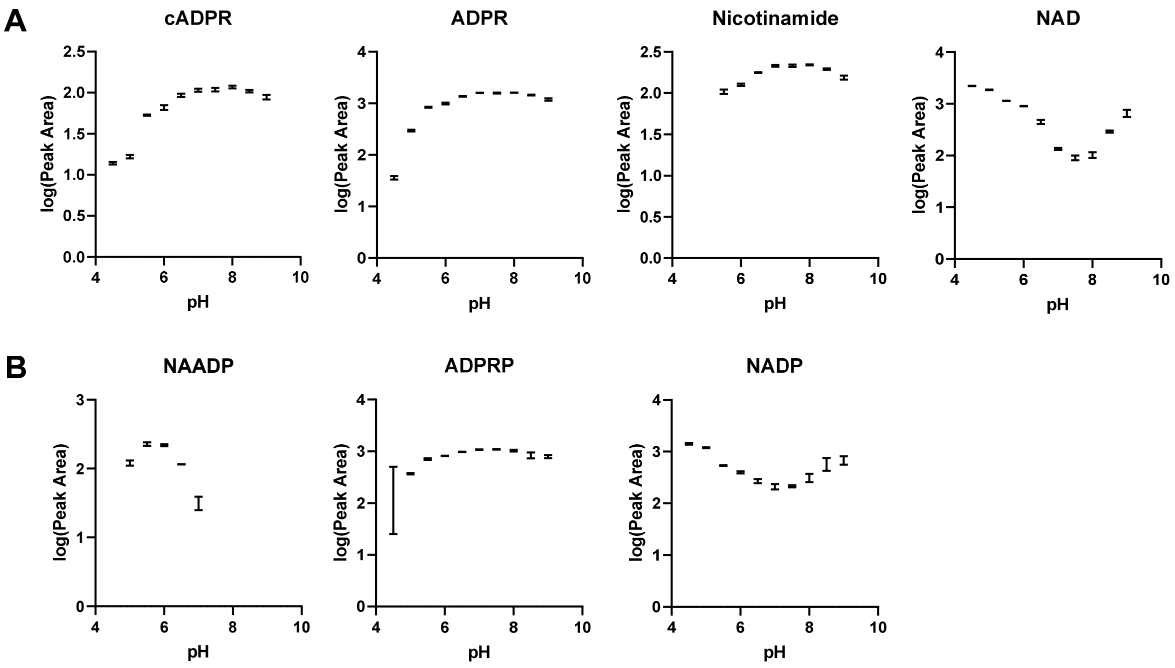

Figure S1. A) pH profile for NAD^+ hydrolysis and cyclization. Log(peak areas) from HPLC chromatographs for cADPR, ADPR, and nicotinamide (products) and NAD^+ (substrate) are plotted against pH; n = 3. Error (SD) is plotted but was generally smaller than the size of the symbols so only the SD bars are shown. B) pH profile for the base exchange reaction between $NADP^+$ and nicotinic acid. Log(peak areas) from HPLC chromatographs for NAADP and ADPRP (products) and $NADP^+$ (substrate) are plotted against pH; n = 3. Full chromatograms are depicted in Figure S2.

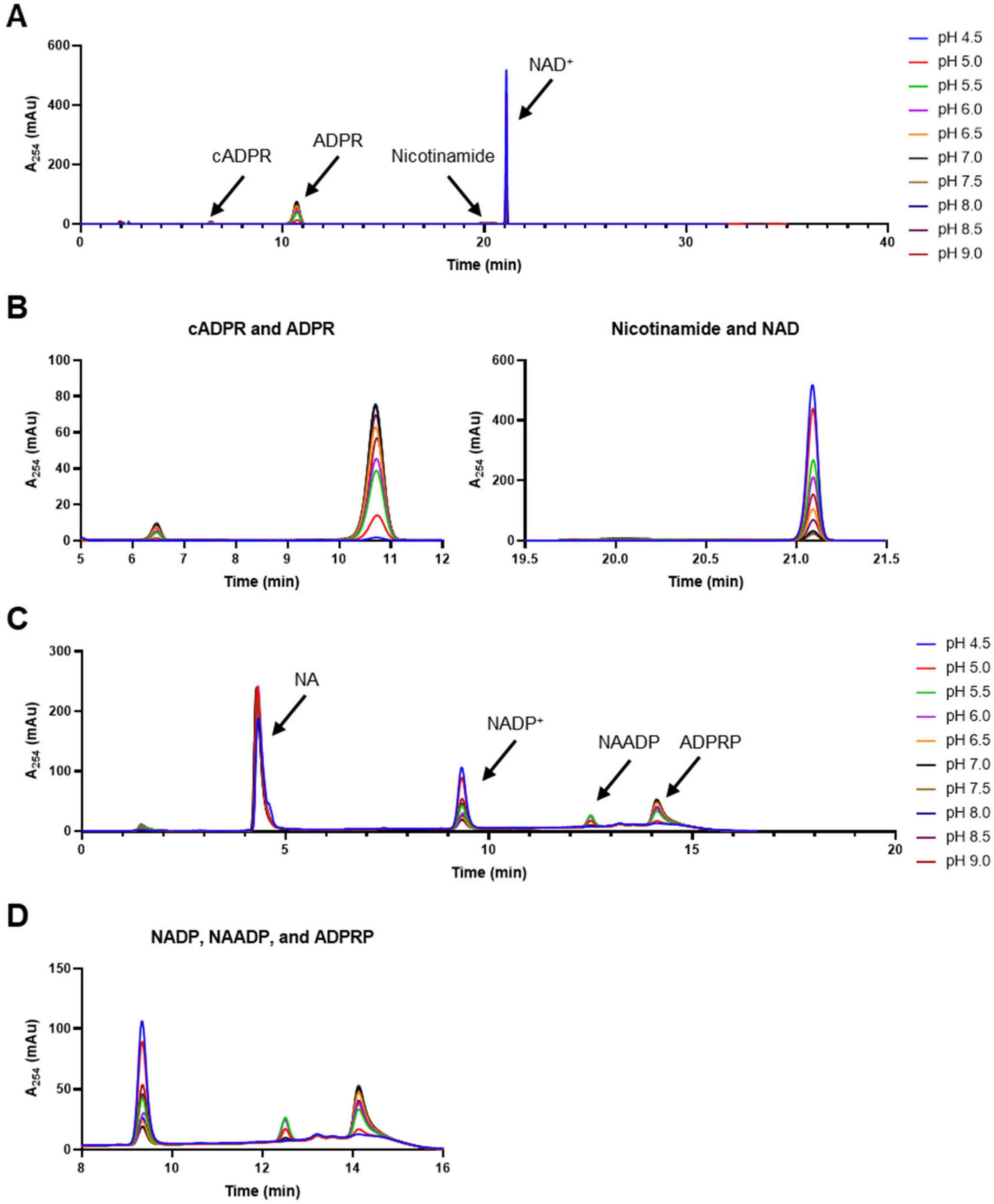

Figure S2. A) Full HPLC chromatograph used in **Fig. S1A**; n = 3, average is shown. B) Blow up of selected regions of the full chromatograph in **Fig. S2A** highlighting the relevant metabolites; n = 3, average is shown. C) Full HPLC chromatograph used in **Fig. S1B**; n = 3, average is shown. D) Blow up of selected regions of the full chromatograph in **Fig. S2C** highlighting the relevant metabolites; n = 3, average is shown.

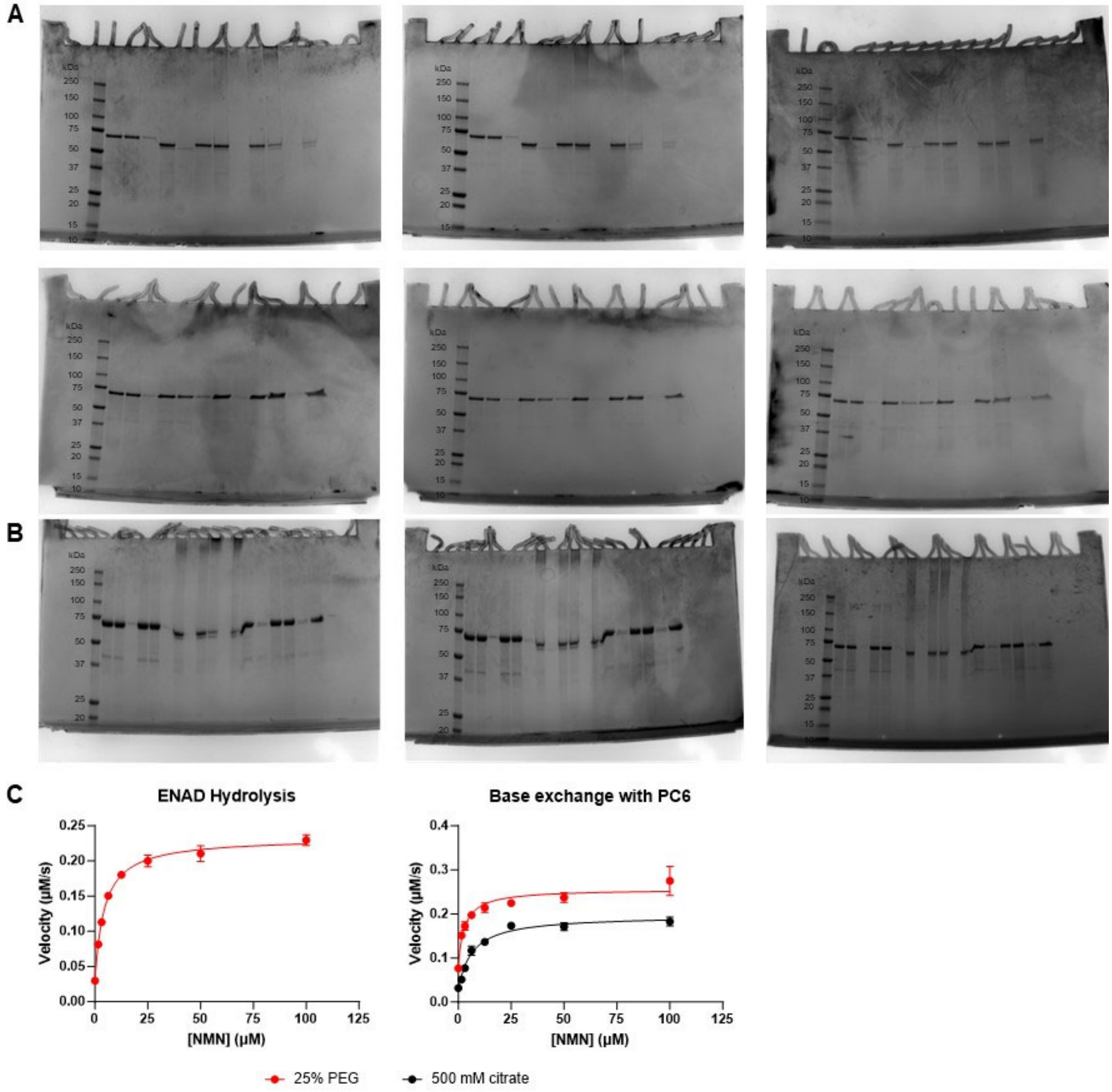

Figure S3. A) Full images of Coomassie stained SDS-PAGE gel in **Fig. 2B;** replicates 1-3 in 25% PEG 3350 (top), replicates 1-3 in 500 mM citrate (bottom). B) Full images of Coomassie stained SDS-PAGE gel in **Fig. 2C;** replicate 1 (left), replicate 2 (middle), replicate 3 (right). C) Expanded NMN concentrations for EC_{50} curves. (Left) ENAD hydrolysis, (right) PC6 base exchange. n = 3. Error bars represent SD.

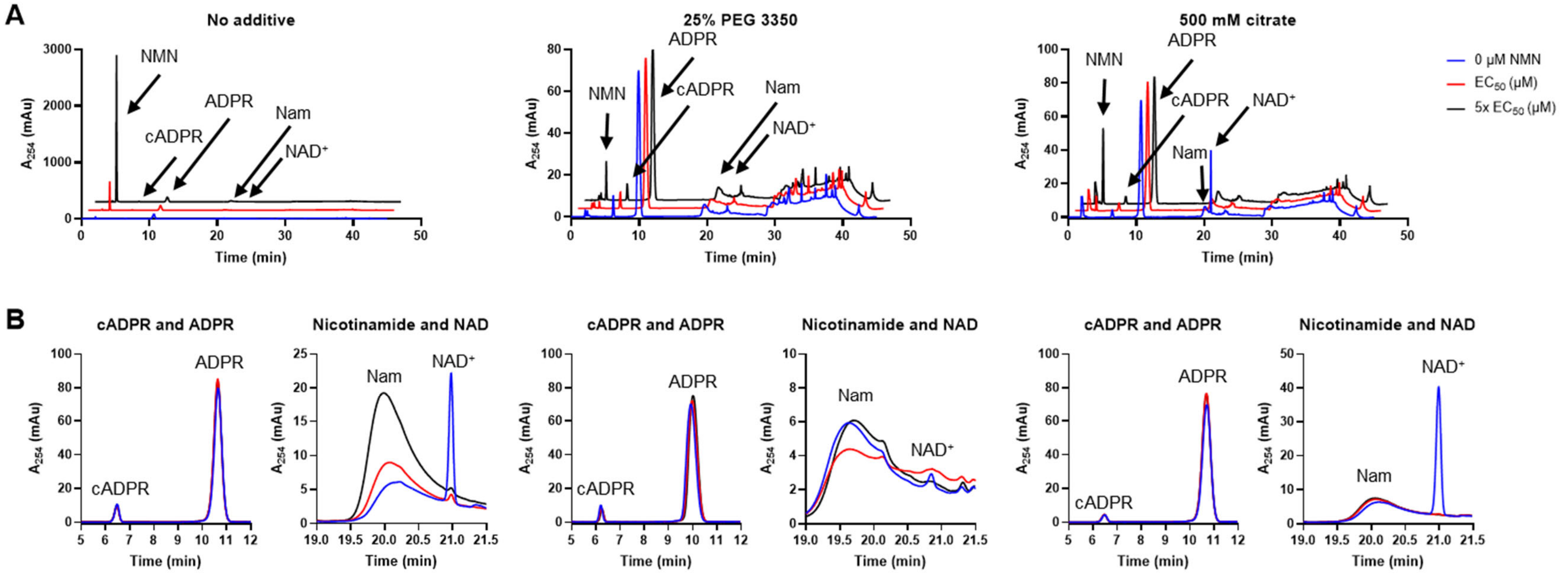

Figure S4. Product specificity for the hydrolysis reaction with NMN and the phase transition. A) Full HPLC chromatographs in **Fig. 4A**; n = 3, average is shown. No additive (left), 25% PEG 3350 (middle), and 500 mM citrate (right). B) Blow up of relevant metabolites in **Fig. S4A**; n = 3, average is shown.

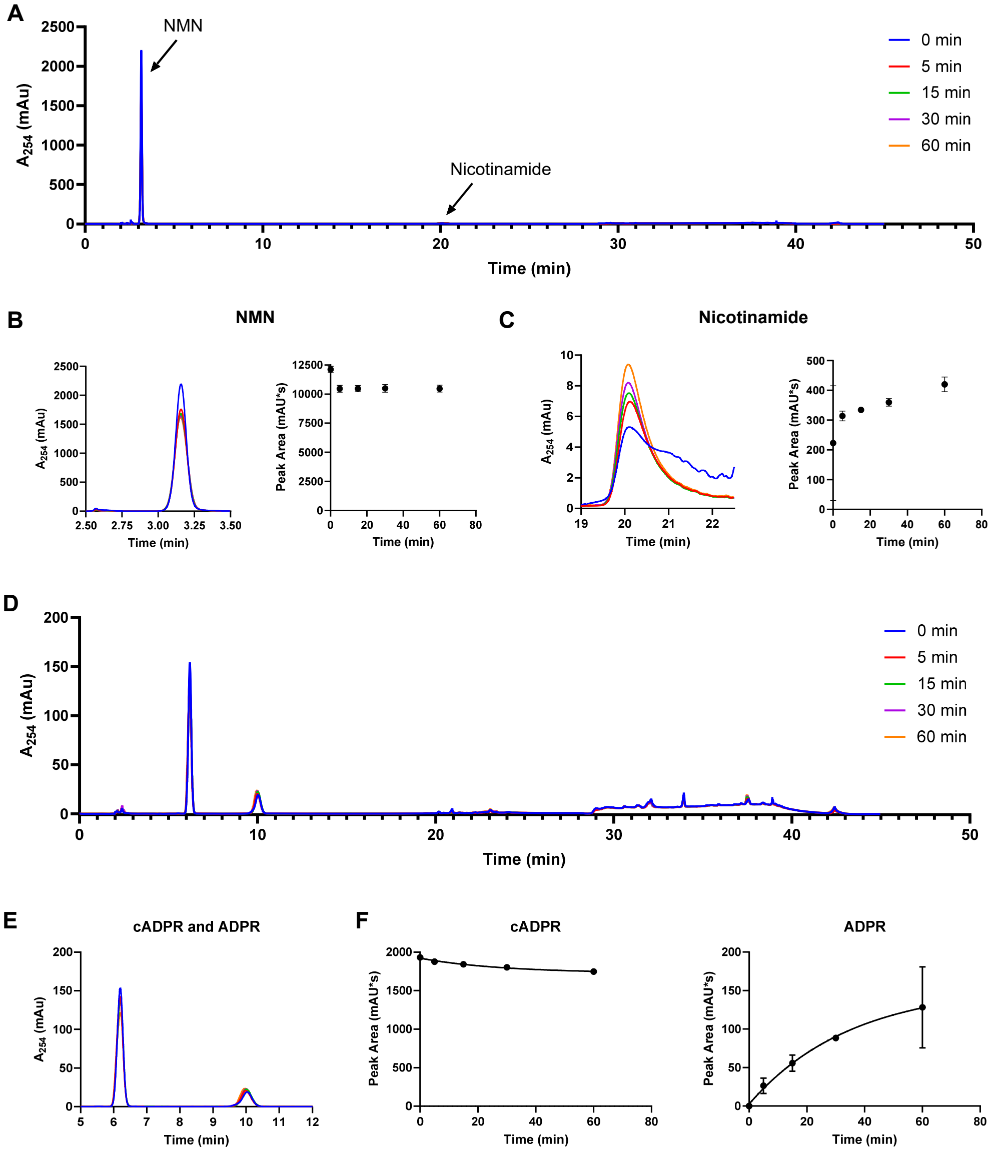

Figure S5. Evaluating NMN and cADPR as SARM1$^{\Delta MLS}$ substrates. A) Evaluation of NMN as a SARM1$^{\Delta MLS}$ substrate by HPLC. Full HPLC chromatograph. B) Blow up of NMN peak (left) and plot of peak area versus time (right). C) Blow up of nicotinamide peak (left) and plot of peak area versus time (right). D) Evaluation of cADPR as a SARM1$^{\Delta MLS}$ substrate by HPLC. Full HPLC chromatograph. E) Blow up of cADPR and ADPR peaks (left) and plot of peak area versus time (right). F) Plots of (c)ADPR peak areas versus time. In B, C, and F, error bars represent SD.

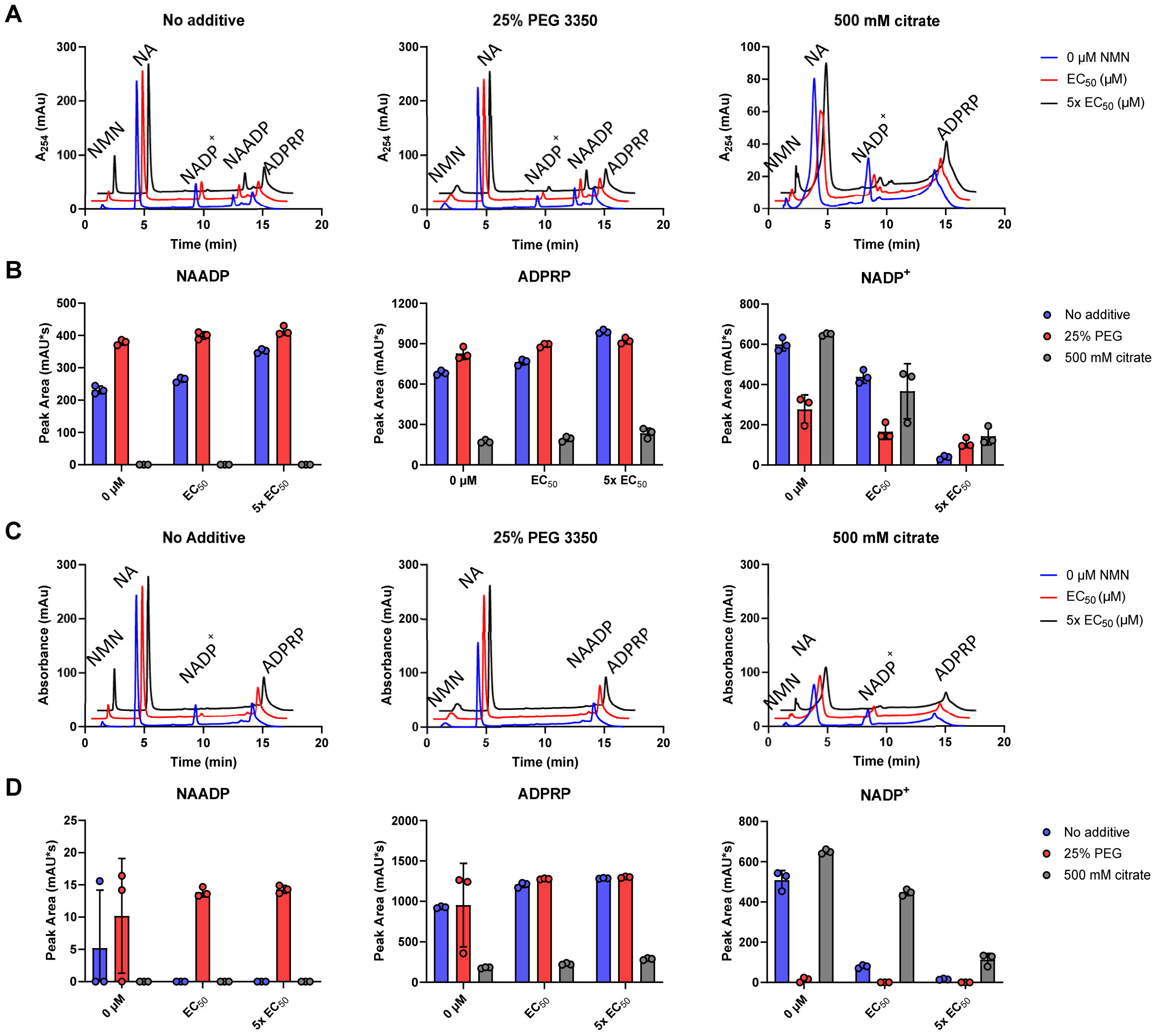

Figure S6. Product specificity of the base exchange reaction with $NADP^+$ and NA after 60 min incubation in the presence or absence of 25% PEG 3350 or 500 mM citrate at 0 µM NMN, NMN at the EC_{50}, and 5x EC_{50}; n = 3. A and B are for experiments at pH 5.5; **C and D** are for experiments at pH 7.5. A) Full HPLC chromatographs. B) Plot of peak areas for NAADP (left), ADPRP (middle), and $NADP^+$ (right). C) Full HPLC chromatographs. D) Plot of peak areas for NAADP (left), ADPRP (middle), and $NADP^+$ (right). In B and D, error bars represent SD.

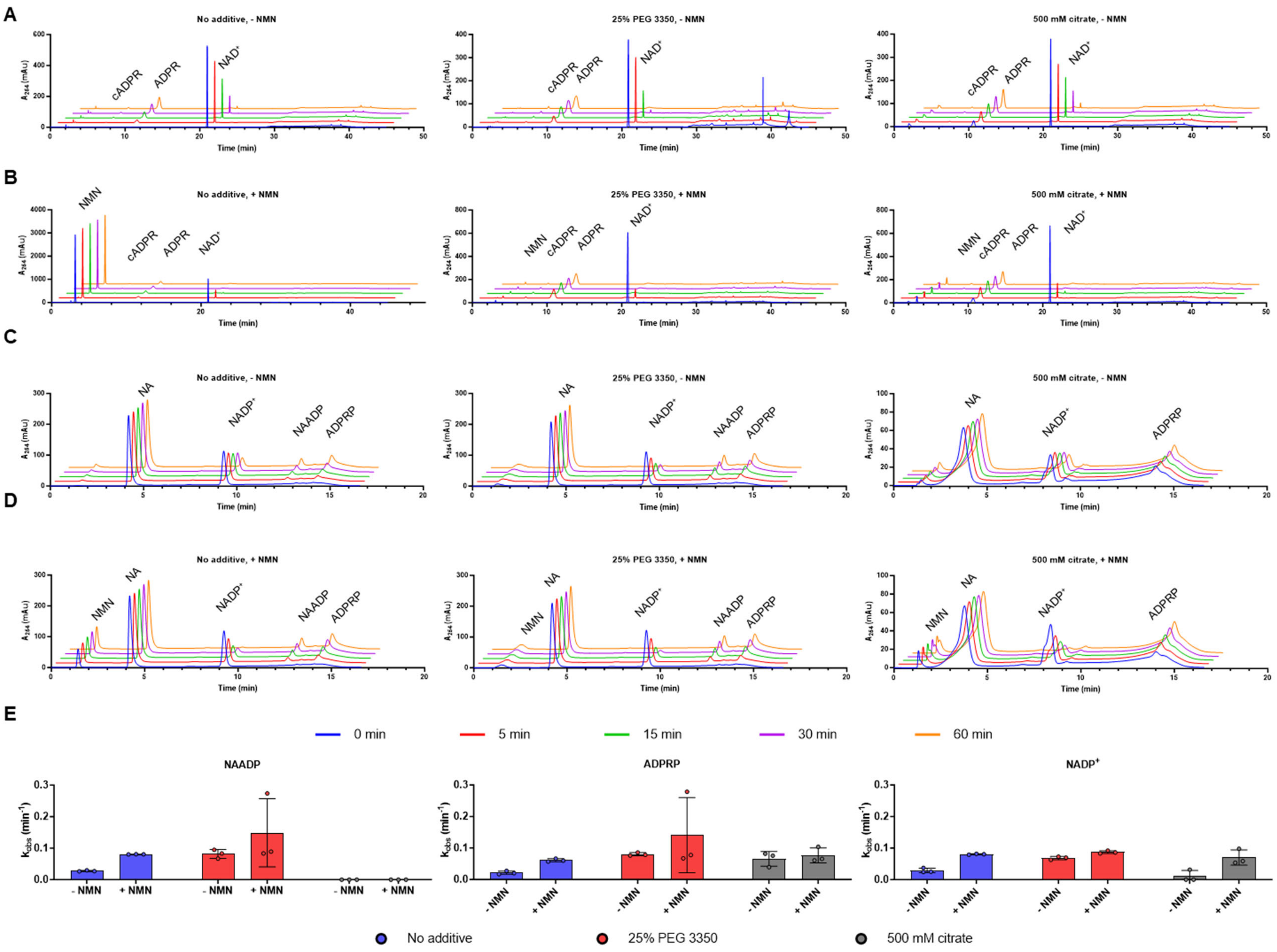
A
No additive, - NMN
25% PEG 3350, - NMN
500 mM citrate, - NMN
B
No additive, + NMN
25% PEG 3350, + NMN
500 mM citrate, + NMN
C
No additive, - NMN
25% PEG 3350, - NMN
500 mM citrate, - NMN
D
No additive, + NMN
25% PEG 3350, + NMN
500 mM citrate, + NMN
NMN
cADPR
ADPR
NAD+
NA
NADP+
NAADP
ADPRP
Time (min)
E
0 min
5 min
15 min
30 min
60 min
NAADP
ADPRP
NADP+
- NMN
+ NMN
No additive
25% PEG 3350
500 mM citrate

Figure S7. Evaluating hydrolysis and base exchange reaction rates with NMN and the phase transition. A) Full HPLC chromatographs for the hydrolysis reaction without NMN in **Fig. 4B**; n = 3, average is shown. No additive (left), 25% PEG 3350 (middle), and 500 mM citrate (right). B) Full HPLC chromatographs for the hydrolysis reaction with NMN in **Fig. 4B**; n = 3, average is shown. No additive (left), 25% PEG 3350 (middle), and 500 mM citrate (right). C) Full HPLC chromatographs for the base exchange reaction without NMN; n = 3, average is shown. No additive (left), 25% PEG 3350 (middle), and 500 mM citrate (right). D) Full HPLC chromatographs for the base exchange reaction with NMN; n = 3, average is shown. No additive (left), 25% PEG 3350 (middle), and 500 mM citrate (right). E) Quantification of time courses in Fig. S7C-D for the base exchange reaction with $NADP^+$ and NA in the presence or absence of 25% PEG 3350 or 500 mM citrate at 0 µM NMN and at EC_{50}; n = 3, error bars reflect SD.

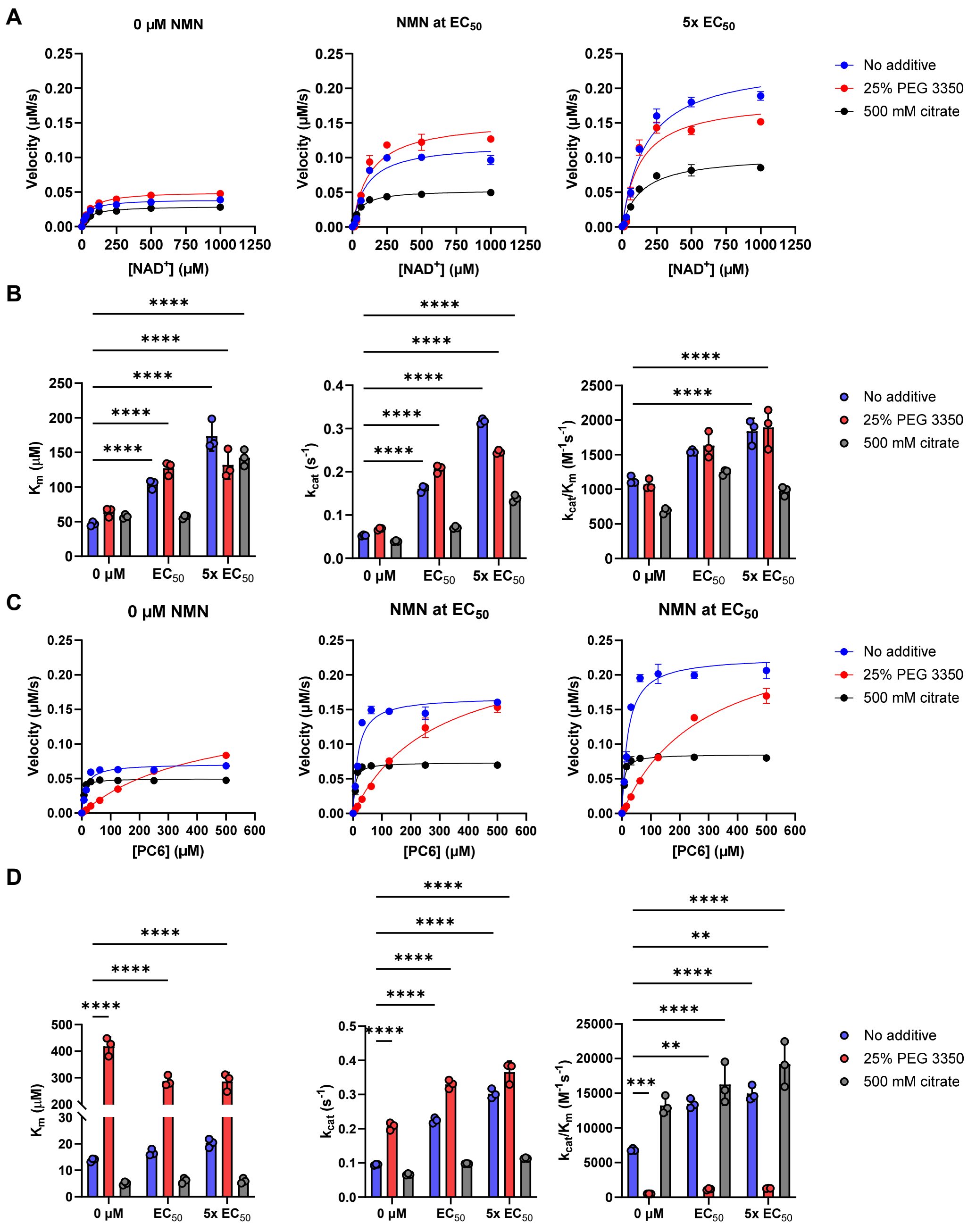

Figure S8. The effect of NMN and the phase transition on base exchange reaction kinetics. A) Kinetic analysis of SARM1$^{\Delta MLS}$ with respect to NAD^+ measured by PC6 base exchange with NAD^+ in the presence or absence of 25% PEG 3350 or 500 mM citrate at 0 µM NMN, NMN at the EC_{50}, and 5x EC_{50}. B) Kinetic parameters for PC6 base exchange with NAD^+ with respect to NAD^+ in the presence or absence of 25% PEG 3350 or 500 mM citrate at 0 µM NMN, NMN at the EC_{50}, and 5x EC_{50}. K_m (left), k_{cat} (middle), k_{cat}/K_m (right). C) Kinetic analysis of SARM1$^{\Delta MLS}$ with respect to PC6 measured by PC6 base exchange with NAD^+ in the presence or absence of 25% PEG 3350 or 500 mM citrate at 0 µM NMN, NMN at the EC_{50}, and 5x EC_{50}. D) Kinetic parameters for PC6 base exchange with NAD^+ with respect to PC6 in the presence or absence of 25% PEG 3350 or 500 mM citrate at 0 µM NMN, NMN at the EC_{50}, and 5x EC_{50}. K_m (left), k_{cat} (middle), k_{cat}/K_m (right). In all cases, error is represented as SD.

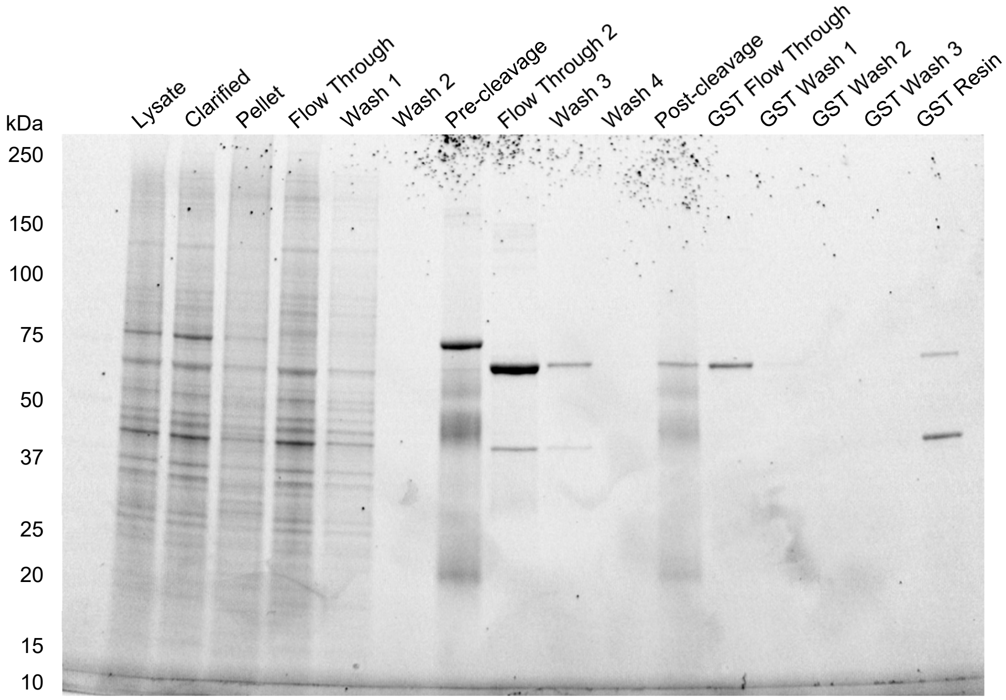

Figure S9. Stain free image of fractions from the purification of SARM1$^{\Delta MLS}$. The GST Flow Through sample is the working material.

Table S1. Molecular crowding techniques used to study the enzymatic activity of SARM1.

Reference	Year	Pure	Molecular crowding technique					
			Lysate	On-Bead	Plates	Forced-dimerization	PEGs/precipitants	Crystallography
**Angeletti et al.**[1]	2022			X				
**Bosanac et al.**[2]	2021		X		X			
**Bratkowski et al.**[3]	2020	X						
**Essuman et al.**[4]	2017			X				
**Essuman et al.**[5]	2018			X				
**Figley et al.**[6]	2021	X						
**Gerdts et al.**[7]	2015					X		
**Gilley et al.**[8]	2021			X				
**Horsefield et al.**[9]	2019		X	X			X	
**Hughes et al.**[10]	2021		X					
**Icso et al.**[11]	2023	X					X	
**Jia et al.**[12]	2022			X				
**Jiang et al.**[13]	2020	X						
**Loring et al.**[14]	2020a		X					
**Loring et al.**[15]	2021						X	
**Loring et al.**[16]	2020b		X					
**Murata et al.**[17]	2018		X	X				
**Peterson et al.**[18]	2022						X	
**Sasaki et al.**[19]	2021			X				
**Shen et al.**[20]	2021			X				
**Shi et al.**[21]	2022	X	X					X
**Sporny et al.**[22]	2020	X						
**Summers et al.**[23]	2016					X		
**Zhao et al.**[24]	2021		X					
**Zhao et al.**[25]	2019	X	X	X				

*These are papers that use recombinant SARM1. This analysis does not include papers where SARM1 activity was evaluated in neurons/cells.

Table S2. HPLC methods used in this paper unless otherwise specified.		
**Substrates**	NAD^+	NA and $NADP^+$
**Column**	Supelcosil LC-18 column (5 µm, 4.6 × 250 mm; Supelco)	POROS HQ column (10 µm, 4.6 × 100 mm, 1.7 mL; ThermoScientific)
**Buffers/Solvents**	**Solvent A:**100 mM potassium phosphate pH 6.0<br>**Solvent B:** 100 mM potassium phosphate pH 6.0 with 20% MeOH<br>**Solvent C:** water with 0.1% formic acid<br>**Solvent D:** acetonitrile with 0.1% formic acid.	**Buffer A:** 10 mM ammonium acetate pH 5.0<br>**Buffer B:** 1 M ammonium acetate pH 5.0
**Gradient details**	• 100% A for 9 min<br>• 0% B to 12% B over 6 min<br>• 12% B to 45% B over 2.5 min<br>• 45% B to 100% over 2.5 min<br>• 100% B for 5.5 min, 100% B to 70% C and 30% D over 1 min<br>• 30% D to 70% D over 8.5 min<br>• 70% D for 1 min<br>• 70% D to 100% A over 5 min<br>• 100% A for 4 min<br><br>Flow rate: 1.3 mL/min	• 100% buffer A for 1 min<br>• 0% to 50% buffer B over 8.25 min<br>• 50% to 100% buffer B over 3 min<br>• 100% buffer B to 100% buffer A over 1 min<br>• 100% buffer A for 3 min<br><br>Flow rate: 1 mL/min

References for supporting information

(S1) Angeletti, C., Amici, A., Gilley, J., Loreto, A., Trapanotto, A. G., Antoniou, C., Merlini, E., Coleman, M. P., and Orsomando, G. (2022) SARM1 is a multi-functional NAD(P)ase with prominent base exchange activity, all regulated bymultiple physiologically relevant NAD metabolites, *iScience 25*, 103812.

(S2) Bosanac, T., Hughes, R. O., Engber, T., Devraj, R., Brearley, A., Danker, K., Young, K., Kopatz, J., Hermann, M., Berthemy, A., Boyce, S., Bentley, J., and Krauss, R. (2021) Pharmacological SARM1 inhibition protects axon structure and function in paclitaxel-induced peripheral neuropathy, *Brain 144*, 3226-3238.

(S3) Bratkowski, M., Xie, T., Thayer, D. A., Brown, S. P., Bai, X., Correspondence, S. S., Lad, S., Mathur, P., Yang, Y.-S., Danko, G., Burdett, T. C., Danao, J., Cantor, A., Kozak, J. A., and Sambashivan, S. (2020) Structural and Mechanistic Regulation of the Pro-degenerative NAD Hydrolase SARM1, *Cell Rep 32*, 107999.

(S4) Essuman, K., Summers, D. W., Sasaki, Y., Mao, X., DiAntonio, A., and Milbrandt, J. (2017) The SARM1 Toll/Interleukin-1 Receptor Domain Possesses Intrinsic NAD(+) Cleavage Activity that Promotes Pathological Axonal Degeneration, *Neuron 93*, 1334-1343.

(S5) Essuman, K., Summers, D. W., Sasaki, Y., Mao, X., Yim, A. K. Y., DiAntonio, A., and Milbrandt, J. (2018) TIR Domain Proteins Are an Ancient Family of NAD(+)-Consuming Enzymes, *Curr Biol 28*, 421-430.

(S6) Figley, M. D., Gu, W., Nanson, J. D., Shi, Y., Sasaki, Y., Cunnea, K., Malde, A. K., Jia, X., Luo, Z., Saikot, F. K., Mosaiab, T., Masic, V., Holt, S., Hartley-Tassell, L., McGuinness, H. Y., Manik, M. K., Bosanac, T., Landsberg, M. J., Kerry, P. S., Mobli, M., Hughes, R. O.,

Milbrandt, J., Kobe, B., DiAntonio, A., and Ve, T. (2021) SARM1 is a metabolic sensor activated by an increased NMN/NAD(+) ratio to trigger axon degeneration., *Neuron 109*, 1118-1136.

(S7) Gerdts, J., Brace, E. J., Sasaki, Y., DiAntonio, A., and Milbrandt, J. (2015) SARM1 activation triggers axon degeneration locally via NAD(+) destruction., *Science 348*, 453-457.

(S8) Gilley, J., Jackson, O., Pipis, M., Estiar, M. A., Gan-Or, Z., Goutman, S. A., Harms, M. B., Kaye, J., Lima, L., Genomics, Q. S., Ravits, J., Rouleau, G. A., Zuchner, S., Reilly, M. M., and Coleman, M. P. (2021) Enrichment of SARM1 alleles encoding variants with constitutively hyperactive NADase in patients with ALS and other motor nerve disorders, *medRxiv*, 211-220.

(S9) Horsefield, S., Burdett, H., Zhang, X., Manik, M. K., Shi, Y., Chen, J., Qi, T., Gilley, J., Lai, J.-S., Rank, M. X., Casey, L. W., Gu, W., Ericsson, D. J., Foley, G., Hughes, R. O., Bosanac, T., von Itzstein, M., Rathjen, J. P., Nanson, J. D., Boden, M., Dry, I. B., Williams, S. J., Staskawicz, B. J., Coleman, M. P., Ve, T., Dodds, P. N., and Kobe, B. (2019) NAD + cleavage activity by animal and plant TIR domains in cell death pathways, *Science 365*, 793-799.

(S10) Hughes, R. O., Bosanac, T., Mao, X., Engber, T. M., DiAntonio, A., Milbrandt, J., Devraj, R., and Krauss, R. (2021) Small Molecule SARM1 Inhibitors Recapitulate the SARM1−/− Phenotype and Allow Recovery of a Metastable Pool of Axons Fated to Degenerate, *Cell Rep 34*, 108588.

(S11) Icso, J. D., Barasa, L., and Thompson, P. R. (2023) SARM1, an Enzyme Involved in Axon Degeneration, Catalyzes Multiple Activities through a Ternary Complex Mechanism, *Biochemistry*.

(S12) Jia, A., Huang, S., Song, W., Wang, J., Meng, Y., Sun, Y., Xu, L., Laessle, H., Jirschitzka, J., Hou, J., Zhang, T., Yu, W., Hessler, G., Li, E., Ma, S., Yu, D., Gebauer, J., Baumann, U., Liu, X., Han, Z., Chang, J., Parker, J. E., and Chai, J. (2022) TIR-catalyzed ADP-ribosylation reactions produce signaling molecules for plant immunity, *Science*.

(S13) Jiang, Y., Liu, T., Lee, C. H., Chang, Q., Yang, J., and Zhang, Z. (2020) The NAD+-mediated self-inhibition mechanism of pro-neurodegenerative SARM1, *Nature 588*, 658-663.

(S14) Loring, H. S., Icso, J. D., Nemmara, V., and Thompson, P. R. (2020) Initial Kinetic Characterization of Sterile Alpha and Toll/Interleukin Receptor Motif-Containing Protein 1., *Biochemistry 59*, 933-942.

(S15) Loring, H. S., Czech, V. L., Icso, J. D., O'Connor, L., Parelkar, S. S., Byrne, A. B., and Thompson, P. R. (2021) A phase transition enhances the catalytic activity of SARM1, an NAD(+) glycohydrolase involved in neurodegeneration, *Elife 10*, e66694.

(S16) Loring, H. S., Parelkar, S. S., Mondal, S., and Thompson, P. R. (2020) Identification of the first noncompetitive SARM1 inhibitors, *Bioorg Med Chem 28*, 115644.

(S17) Murata, H., Khine, C. C., Nishikawa, A., Yamamoto, K. i., Kinoshita, R., and Sakaguchi, M. (2018) C-Jun N-terminal kinase (JNK)-mediated phosphorylation of SARM1 regulates NAD cleavage activity to inhibit mitochondrial respiration, *Journal of Biological Chemistry 293*, 18933-18943.

(S18) Peterson, N. D., Icso, J. D., Salisbury, J. E., Rodriguez, T., Thompson, P. R., and Pukkila-Worley, R. (2022) Pathogen infection and cholesterol deficiency activate the C. elegans p38 immune pathway through a TIR-1/SARM1 phase transition, *eLife 11*, e74206.

(S19) Sasaki, Y., Zhu, J., Shi, Y., Gu, W., Kobe, B., Ve, T., Diantonio, A., and Milbrandt, J. (2021) Nicotinic acid mononucleotide is an allosteric SARM1 inhibitor promoting axonal protection, *Experimental Neurology 345*, 113842.

(S20) Shen, C., Vohra, M., Zhang, P., Mao, X., Figley, M. D., Zhu, J., Sasaki, Y., Wu, H., DiAntonio, A., and Milbrandt, J. (2021) Multiple domain interfaces mediate SARM1 autoinhibition, *Proceedings of the National Academy of Sciences 118*, 1-9.

(S21) Shi, Y., Kerry, P. S., Nanson, J. D., Bosanac, T., Sasaki, Y., Krauss, R., Saikot, F. K., Adams, S. E., Mosaiab, T., Masic, V., Mao, X., Rose, F., Vasquez, E., Furrer, M., Cunnea, K., Brearley, A., Gu, W., Luo, Z., Brillault, L., Landsberg, M. J., Di Antonio, A., Kobe, B., Milbrandt, J., Hughes, R. O., and Ve, T. (2022) Structural basis of SARM1 activation, substrate recognition, and inhibition by small molecules, *Mol Cell 82*, 1643-1659.

(S22) Sporny, M., Guez-Haddad, J., Khazma, T., Yaron, A., Dessau, M., Shkolnisky, Y., Mim, C., Isupov, M. N., Zalk, R., Hons, M., and Opatowsky, Y. (2020) Structural basis for SARM1 inhibition and activation under energetic stress, *eLife 9*, e62021.

(S23) Summers, D. W., Gibson, D. A., DiAntonio, A., and Milbrandt, J. (2016) SARM1-specific motifs in the TIR domain enable NAD+ loss and regulate injury-induced SARM1 activation, *Proceedings of the National Academy of Sciences of the United States of America 113*, E6271-E6280.

(S24) Zhao, Y. J., He, W. M., Zhao, Z. Y., Li, W. H., Wang, Q. W., Hou, Y. N., Tan, Y., and Zhang, D. (2021) Acidic pH irreversibly activates the signaling enzyme SARM1, *FEBS Journal 288*, 6783-6794.

(S25) Zhao, Z. Y., Xie, X. J., Li, W. H., Liu, J., Chen, Z., Zhang, B., Li, T., Li, S. L., Lu, J. G., Zhang, L., Zhang, L. h., Xu, Z., Lee, H. C., and Zhao, Y. J. (2019) A Cell-Permeant Mimetic of NMN Activates SARM1 to Produce Cyclic ADP-Ribose and Induce Non-apoptotic Cell Death, *iScience 15*, 452-466.
